# Effects of Salt Stress on Transcriptional and Physiological Responses in Barley Leaves with Contrasting Salt Tolerance

**DOI:** 10.3390/ijms23095006

**Published:** 2022-04-30

**Authors:** Rim Nefissi Ouertani, Dhivya Arasappan, Tracey A. Ruhlman, Mariem Ben Chikha, Ghassen Abid, Samiha Mejri, Abdelwahed Ghorbel, Robert K. Jansen

**Affiliations:** 1 Laboratory of Plant Molecular Physiology, Center of Biotechnology of Borj Cedria, BP 901, Hammam-Lif 2050, Tunisia; mariam.tn@gmx.fr (M.B.C.); mejrisamiha2018@gmail.com (S.M.); wahidghorbel@yahoo.fr (A.G.); 2 Center for Biomedical Research Support, University of Texas at Austin, Austin, TX 78712, USA; darasappan@austin.utexas.edu; 3 Department of Integrative Biology, University of Texas at Austin, Austin, TX 78712, USA; truhlman@austin.utexas.edu; 4 Laboratory of Legumes and Sustainable Agrosystems, Center of Biotechnology of Borj Cedria, BP 901, Hammam-Lif 2050, Tunisia; abidghassen@gmail.com; 5 Biotechnology Research Group, Department of Biological Sciences, Faculty of Science, King Abdulaziz University (KAU), Jeddah 21589, Saudi Arabia

**Keywords:** *Hordeum vulgare* L., landrace, salt tolerance, photosynthesis, antioxidant enzymes, RNA-seq, differentially expressed genes, co-expression network

## Abstract

Salt stress negatively impacts crop production worldwide. Genetic diversity among barley (*Hordeum vulgare*) landraces adapted to adverse conditions should provide a valuable reservoir of tolerance genes for breeding programs. To identify molecular and biochemical differences between barley genotypes, transcriptomic and antioxidant enzyme profiles along with several morpho-physiological features were compared between salt-tolerant (Boulifa) and salt-sensitive (Testour) genotypes subjected to salt stress. Decreases in biomass, photosynthetic parameters, and relative water content were low in Boulifa compared to Testour. Boulifa had better antioxidant protection against salt stress than Testour, with greater antioxidant enzymes activities including catalase, superoxide dismutase, and guaiacol peroxidase. Transcriptome assembly for both genotypes revealed greater accumulation of differentially expressed transcripts in Testour compared to Boulifa, emphasizing the elevated transcriptional response in Testour following salt exposure. Various salt-responsive genes, including the antioxidant catalase 3, the osmoprotectant betaine aldehyde dehydrogenase 2, and the transcription factors *MYB20* and *MYB41*, were induced only in Boulifa. By contrast, several genes associated with photosystems I and II, and light receptor chlorophylls A and B, were more repressed in Testour. Co-expression network analysis identified specific gene modules correlating with differences in genotypes and morpho-physiological traits. Overall, salinity-induced differential transcript accumulation underlies the differential morpho-physiological response in both genotypes and could be important for breeding salt tolerance in barley.

## 1. Introduction

Barley (*Hordeum vulgare* L.) is the fourth most economically important cereal crop worldwide in terms of both quantity and area under cultivation [1]. A versatile crop, barley is mainly used for feed and industry but also has great potential as healthy food source due to an abundance of dietary fiber and functional food constituents [2]. Domesticated thousands of years ago, barley is grown in a wide range of geographic and climatic conditions [3], reflecting high adaptability facilitated by genetic diversity [4]. Like most crops, barley development, productivity, and yield are impaired by environmental stresses such salinity. Soil salinity is recognized as a major environmental stress [5], and ever-changing global climatic factors amplify its effects. Salinity-induced plant responses include hyper-osmotic stress, ion toxicity due to imbalance of cellular ion homeostasis, nutritional imbalance, and oxidative damage due to the excessive generation of reactive oxygen species (ROS) [5,6,7].

Among cereals, barley is considered salt-tolerant [8,9], characterized by great variation in tolerance among cultivars [10]. Salinity tolerance in barley is a very complex process that involves the interaction of diverse regulatory pathways including water uptake and osmotic tolerance via osmoprotectant biosynthesis, photosynthesis regulation, hormone signaling, ion homeostasis adjustment, and antioxidant metabolism. All of these pathways are activated across complex salinity-sensing and signaling networks along with members of several stress-related gene expression regulator families [5].

Evolving in regions with marginal conditions, barley landraces adapted to harsh environments could provide a reservoir of tolerance alleles [11]. Therefore, exploration of landrace cultivars that exhibit contrasting responses toward salinity is of great interest for elucidating candidate genes and tolerance mechanisms. Such information could guide breeding programs that aim to enhance yields under stressful conditions.

In recent years, next-generation nucleic acid sequencing, including RNA sequencing (RNA-seq), has been widely employed in many plant species. This technology has been used to identify polymorphisms, characterize transcript populations, and detect differential gene expression networks between varieties/genotypes that demonstrate variable levels of stress tolerance [4,12]. These studies have allowed an improved understanding of the regulatory networks involved in plant stress responses and suggest avenues to increase tolerance and plant productivity [13].

The transcriptomic approach has been used to develop markers associated with salt tolerance. Several studies have focused on barley [14,15,16,17]. Other groups examined wheat (*Triticum aestivum*) [18,19] and maize [20] (*Zea mays*) RNA-seq data. Various studies have underscored the importance of analyzing transcriptomes from genotypes differing in stress tolerance in order to better understand stress tolerance processes in barley. Variation in root-specific transcriptional responses to salt stress were observed between barley genotypes that displayed contrasting tolerance phenotypes [21] and differential responses to drought and heat stress were identified between barley cultivars [22]. Moreover, sequencing of two wild barley accessions with contrasting drought tolerance revealed genotype-specific transcripts [23,24,25]. Several research studies have explored the physiological responses of barley to salt stress [26,27,28,29,30,31,32]. Studies have combined both physiological and transcriptomic approaches to examine drought [22,25] and heat stress responses in barley [22]; salt tolerance in rice [33] and drought stress response in maize [34]. Nevertheless, there is a lack of information about associations between physiological and transcriptomic responses to salt stress in barley. Therefore, it is of great interest to connect RNA-seq data to observed physiological changes induced by salt stress in contrasting barley genotypes.

Barley cultivars Boulifa (B; salt-tolerant) and Testour (T; salt-sensitive) were identified in a screening of 21 accessions that represent the genetic diversity among barley landraces in Tunisia [35]. Herein, these two barley genotypes, which demonstrate contrasting salinity tolerance, were subjected to severe salt stress (200 mM) and evaluated at different durations of exposure. Stress-response properties were investigated at both transcriptional and physiological levels. Transcriptomes of both tolerant and sensitive genotypes were sequenced, and gene expression changes were evaluated. Molecular functions, biological processes, and co-expression networks of salt-modulated genes are reported. Overall, the RNA-seq data demonstrated that B and T transcription profiles responded differentially to salt stress. These differences underlie the distinctive phenotypic plasticity of barley genotypes in response to salinity.

## 2. Results

### 2.1. Morphological and Physiological Responses of Barley Genotypes under Salt Stress

**Figure 1:**
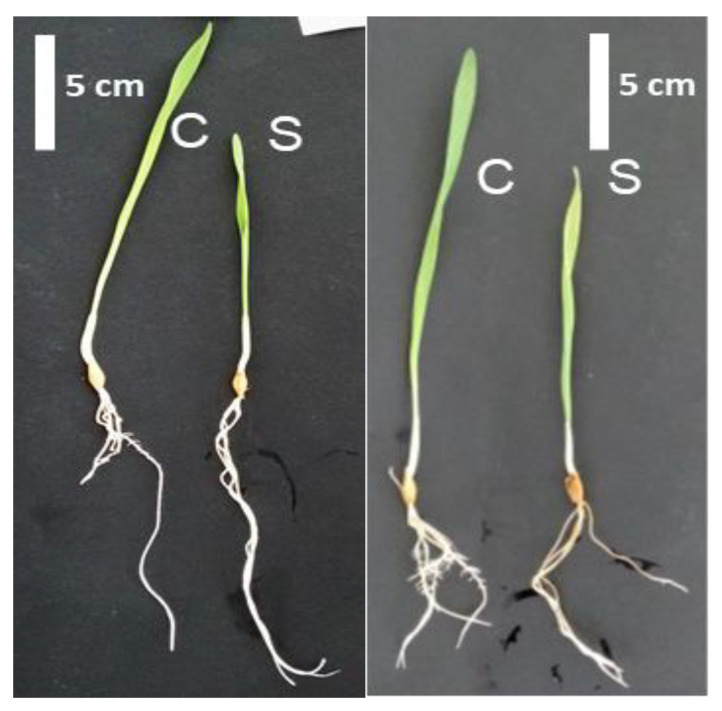
Phenotype of Boulifa (B, **right**) and Testour (T, **left**) grown under control conditions (C) and salt (200 mM NaCl) treatment (S) for 24 h.

Two barley genotypes with differences in tolerance to salt stress were subjected to 200 mM NaCl for 24 h in a hydroponic culture system to assess morphological and physiological differences. At the end of treatment, both cultivars B and T exhibited a decrease in growth relative to their respective controls (Figure 1). Decreases of all measured parameters, fresh weight (FW), dry weight (DW), and shoot length (L), were more pronounced in the salt-sensitive genotype T relative to the salt-tolerant B (Table 1). Compared to within genotype controls DW decreased by 35.5% in T, whereas a 12.8% decline was measured in B (Table 1).

In response to salt stress, the relative water content (RWC) was significantly reduced in both genotypes compared to their corresponding controls and a smaller reduction in RWC was recorded for the tolerant B (4.5%) than the sensitive T (7.2%). With respect to osmotic potential (OP), the average decrease relative to controls was similar in both B and T with an average value of 17% (Table 1).

**Table 1:** Growth parameters in leaves of control and salt-stressed seedlings.

Genotype	Treatment	FW	DW	L	RWC	OP
Boulifa	Control	201.76 ± 1.1 ^b^	15.80 ± 0.2 ^b^	17.06 ± 0.75 ^b^	0.97 ± 0.04 ^a^	−1.03 ± 0.05 ^b^
	Salt stress	184.60 ± 1.6 ^c^	13.76 ± 0.7 ^c^	16.26 ± 0.40 ^b,c^	0.92 ± 0.02 ^b^	−1.21 ± 0.12 ^a^
Testour	Control	252.80 ± 0.9 ^a^	21.23 ± 0.8 ^a^	19.66 ± 1.10 ^a^	0.96 ± 0.08 ^a^	−1.03 ± 0.03 ^b^
	Salt stress	198.36 ± 1.1 ^b^	13.70 ± 1.2 ^c^	14.50 ± 0.81 ^c^	0.89 ± 0.02 ^c^	−1.20 ± 0.05 ^a^

Data are mean ± SE of three replicates. Different lower-case letters (a, b, c) indicate that means in the same column with different superscripts are significantly different (Tukey’s HSD, *p* ≤ 0.05). Fresh weight (FW; mg), Dry weight (DW; mg), shoot length (L; cm), relative water content (RWC; %), osmotic potential (OP; Mpa).

Photosynthetic characteristics (net CO_2_ assimilation rate (Pn), stomatal conductance (Gs), transpiration rate (E), internal concentration of CO_2_ (Ci), and water-use efficiency (WUE)) were significantly decreased in both genotypes following 24 h of salt treatment (Table 2). Nevertheless, the tolerant genotype B had consistently higher Pn, Gs, E, Ci, and WUE than T. Declines in Pn were 45% and 30% of the control in T and B, respectively. A similar trend was observed for Gs (50% and 20% of the control in T and B, respectively). For E and Ci the effect of salt stress was less pronounced. In the salt-treated B, parameters E and Ci decreased by an average of 12% of control whereas in T this reduction was 31% and 20%, respectively. Relative to the respective controls, WUE decline was similar in both B and T genotype (~20%).

The photosynthetic pigment chlorophyll a (Chl a) was reduced by approximately 25% in both genotypes under 200 mM NaCl compared to their respective controls (Table 2). While chlorophyll b (Chl b) and Chl a were similarly reduced in genotype B, i.e., 25%, genotype T experienced a 42% reduction for Chl b, significantly different than Chl a in the T genotype (25%).

**Table 2:** Effect of salt stress on photosynthetic parameters in leaves of control and salt-stressed seedlings of Boulifa and Testour.

Genotype	Treatment	Pn	Gs	E	Ci	WUE	Chl a	Chl b
Boulifa	Control	7.67 ± 0.27 ^b^	0.06 ± 0.00 ^b^	0.55 ± 0.03 ^c^	236.33 ± 1.5 ^a^	14.00 ± 1.33 ^a^	4.64 ± 0.04 ^b^	1.99 ± 0.02 ^b^
	Salt stress	5.39 ± 0.24 ^c^	0.05 ± 0.01 ^c^	0.47 ± 0.01 ^d^	210.66 ± 1.5 ^b^	11.32 ± 0.81 ^b^	3.62 ± 0.75 ^c^	1.45 ± 0.28 ^c^
Testour	Control	9.35 ± 0.52 ^a^	0.08 ± 0.02 ^a^	0.91 ± 0.06 ^a^	171.33 ± 1.2 ^c^	10.24 ± 0.97 ^b^	6.43 ± 1.21 ^a^	2.69 ± 0.72 ^a^
	Salt stress	5.15 ± 0.10 ^c^	0.04 ± 0.00 ^c^	0.63 ± 0.05 ^b^	135.33 ± 0.9 ^d^	8.17 ± 0.69 ^c^	4.85 ± 0.77 ^c^	1.56 ± 0.36 ^c^

Data are mean ± SE of three replicates. Different lower-case letters (a, b, c, d) indicate that means in the same column with different superscripts are significantly different (Tukey’s HSD, *p* ≤ 0.05). Net CO_2_ assimilation rate (Pn; μmol CO_2_ m^−2^ s^−1^), stomatal conductance (Gs; mol H_2_O m^−2^ s^−1^), transpiration rate (E; mmol H_2_O m^−2^ s^−1^), internal concentration of CO_2_ (Ci; μmol CO_2_ mol^−1^), and water use efficiency (WUE: nmol CO_2_ mol^−1^ H_2_O) and Chlorophyll a/b content (Chl and Chl b; mg/g MF).

### 2.2. Antioxidant Enzyme Responses to Salt Stress in Leaves and Roots

Differential activity of antioxidant enzymes between the barley genotypes was assessed. Changes in the major antioxidant enzymes superoxide dismutase (SOD), catalase (CAT), ascorbate peroxidase (APX), guaiacol peroxidase (GPX), and glutathione reductase (GR) were analyzed in salt-stressed B and T early in development, 12 days after emergence. Salt exposure (200 mM, 24 h) induced oxidative stress in both genotypes as indicated by increased activity of antioxidant enzymes relative to respective controls, although overall the increases were greater in B than T (Table 3). When exposed to salt, SOD activity was elevated to a greater extent in B (50%) than in T (20%) compared to respective controls (Table 3). Similar trends were observed for CAT and GPX enzymes. Regarding APX and GR, no significant changes were detected for either genotype compared to their respective controls, even though a slight increase was noted in APX activity in T genotype (Table 3).

### 2.3. RNA-seq Analysis of Salt Response in Barley Genotypes

RNA sequencing of three replicates for each salt stress treatment (0, 2, 8, and 24 h) resulted in an average of 23.9 million raw reads per sample and 62.5%, on average, of the reads from each sample aligned to the barley transcriptome and led to the identification of 32,943 genes (Table S1).

Principal Component Analysis (PCA) and hierarchical clustering were performed using 500 genes with the largest variance in expression to visualize the underlying structure of the RNA-seq data and assess the largest source of variation among the samples.

**Figure 2:**
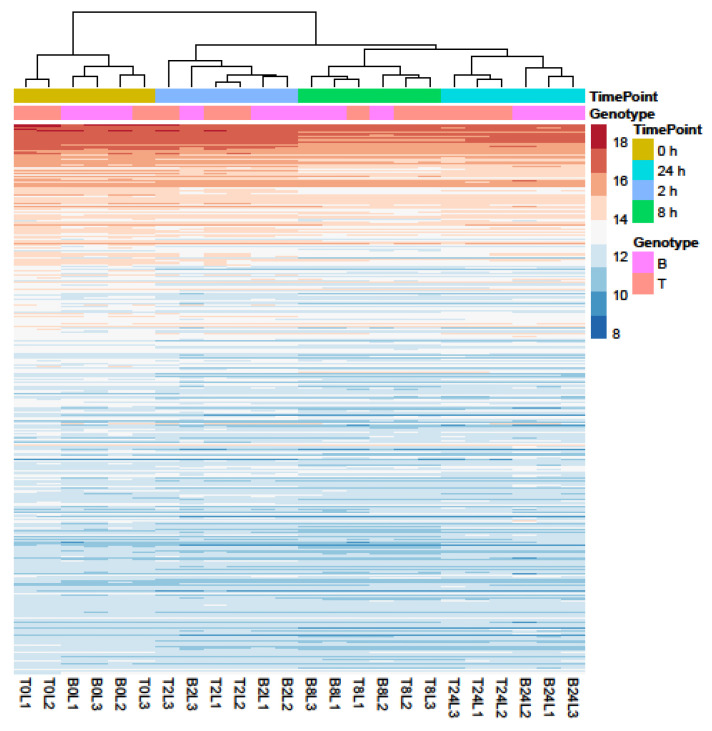
Profiles of the 500 genes with the greatest variance in expression. Data were collected over four durations (0, 2, 8, and 24 h) of salt treatment in Boulifa (B, pink) and Testour (T, orange) seedlings. The colors indicate expression level of genes (see key on right). Sampling time points and genotypes (L1, L2, and L3) are shown above cluster plot. Biological replicates are labeled on the *x*-axis. The dendrogram (top) indicates sample clustering.

**Figure 3:**
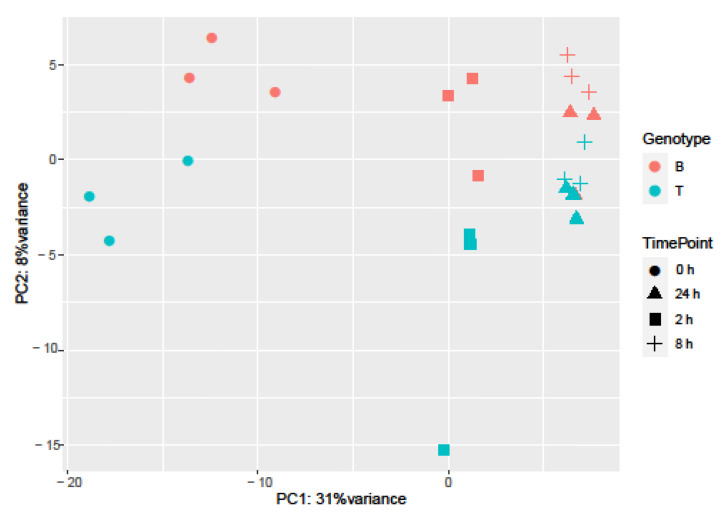
Sources of variation in barley gene expression under salt stress. Principal component analysis was employed to explore influences on variation. Genotype is represented by color (Boulifa—orange; Testour—blue) and treatment is represented by shape (0, 2, 8, and 24 h salt stress, see key at right).

Samples separated into two major clusters of control (0 h) and salt-treated samples (2, 8, and 24 h) (Figure 2). The salt-stressed samples also separated according to the duration of exposure, though this signal was less distinct between 8 and 24 h salt treatments. Clustering of the genotypes was less prominent but replicates of both controls and salt-treated samples of B and T for the most part remained distinct (Figure 2 and Figure 3). In the PCA, the largest source of variation (31%) represented by the *x*-axis corresponds to controls (0 h) vs. salt stress (2, 8, and 24 h salt stress). The second largest source of variation (8%) represented by the *y*-axis may correspond to differences in genotype. According to PCA (Figure 3) and clustering (Figure 2), 24 h salt stress clustered the farthest from control samples (0 h), indicating that the duration of salt treatment was likely the largest source of variation in the data. Therefore, further bioinformatic analyses and correlation with physiological data were conducted on controls (0 h) and 24 h salt-treated samples of both genotypes.

### 2.4. Differentially Expressed Genes in Boulifa and Testour in Response to Salt Stress

**Figure 4:**
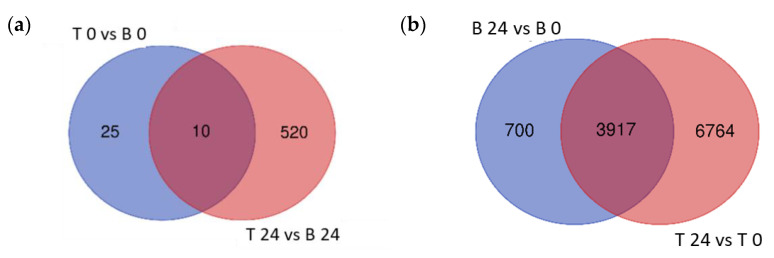
Venn diagrams of the differentially expressed genes. Differentially expressed genes (DEGs) of the different contrast groups ((**a**) B 24 vs. B 0 and T 24 vs. T 0) and ((**b**) T 0 vs. B 0 and T 24 vs. B 24) were enumerated to illustrate the specific DEGs for each comparison and their overlap.

To explore the variation in transcriptional response between B and T genotypes in relation to salt stress and between control (0 h) and salt stress (24 h) treatments in each genotype, differential gene expression analysis was conducted for four contrasting groups (T 0 vs. B 0, T 24 vs. B 24, B 24 vs. B 0, and T 24 vs. T 0). Based on relative transcript abundance, differentially expressed genes (DEGs) were characterized as upregulated (↑) or downregulated (↓). The greatest number of DEGs was found between T 24 vs. T 0 (10,681:8362 ↑ and 3126 ↓; Figure 4a red) followed by B 24 vs. B 0 (4617:2528 ↑ and 2170 ↓; Figure 4a blue). These two contrast groups shared a high number of DEGs (3917; Figure 4a); only 700 DEGs were specific to B 24 vs. B 0. The elevated number of specific DEGs of T 24 vs. T 0 (6764), indicated that T activated a more robust transcriptional response than B at 24 h of salt stress treatment.

The lowest number of total DEGs, just 35 (11 ↑ and 24 ↓), was detected in the contrast between both analyzed genotypes under control conditions (T 0 vs. B 0; Figure 4b). Given that B and T arose in the same geographical location and likely share some genetic similarity, this was expected. However, after 24 h salt treatment, 530 DEGs were identified between T and B genotypes (362 ↑ and 168 ↓), indicating that the salt-induced transcriptomic responses were genotype specific. Furthermore, the number of T specific DEGs (520) was 20 times higher than B specific DEGs (25), which likely contributed to the differential responses to salt stress between these two genotypes and supported the morphological and physiological variation detected (Figure 4).

### 2.5. Gene Ontology Enrichment Analysis of DEGs

To further elucidate the regulatory variation in biological processes and molecular functions between both salt-stressed genotypes, DEGs identified from the four contrast groups (B 24 vs. B 0, T 24 vs. T 0, T 0 vs. B 0, and T 24 vs. B 24) were subjected to gene ontology (GO) enrichment analysis.

**Figure 5:**
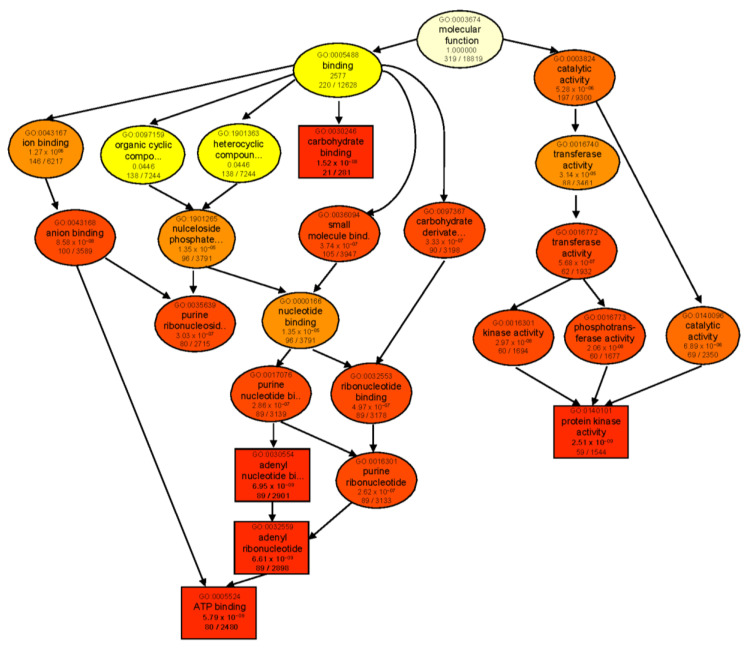
GO enrichment analysis of DEGs of the contrast group T 24 vs. B 24. Rectangles/circles contain the GO term number and category name. The numerals at the bottom in each box are the *p*-value followed by the number of genes in the input list that overlapped with a given GO term over the total number of genes in the GO term. The colors indicate levels of statistical significance, with darker colors indicating a more significant level of enrichment. The rectangles indicate top five enriched terms.

When comparing each genotype against its respective control, ‘catalytic activity’ was the most significantly enriched molecular function category in both genotypes (Figure S1a,b). In B, enriched DEGs were mainly involved in lyase, transferase, glycosyltransferase, and sucrose synthase activities. However, in T, catalytic activity was mainly represented by GO terms for ligase and aminoacyl−tRNA ligase activities. GO enrichment analysis of genotype-dependent DEGs under control conditions (T 0 vs. B 0) also revealed ‘catalytic activity’ as the main enriched category (Figure S1c). However, after 24 h salt treatment (T 24 vs. B 24), many more GO categories were enriched with the major identified subcategories of ‘binding’ and ‘catalytic activity’. For binding activity, several types of ion-, anion-, carbohydrate-, small-molecule-, and ATP-binding proteins were found. As for ‘catalytic activity’, transferase, kinase, and phosphotransferase activities were the most prominent GO terms in both genotypes following salt treatment (Figure 5).

Regarding biological processes, when comparing each salt-stressed genotype to its respective control, the major annotated categories were metabolic and cellular processes. For cellular process, protein folding was the most prominent in both B and T after 24 h salt treatment. However, for metabolic process the dominant annotated subcategories were different between B 24 vs. B 0 and T 24 vs. T 0. For B 24 vs. B 0, metabolic process subcategories were the predominant including carbohydrate metabolic, specifically, sucrose metabolism. Additionally, processes vital to photosynthesis were indicated. Dominant were organic substance metabolic processes such as tetrapyrrole and porphyrin−containing biosynthesis. Among the DEGs indicated, those ↑ respond to abiotic stress including oxygen-containing compounds, to water, and organonitrogen compounds.

For T 24 vs. T 0, the biological process pattern was much more complicated than in the B 24 vs. B 0 comparison. Over-represented categories included small molecule, organic substance, and cellular metabolic processes leading to tRNA aminoacylation, and porphyrin-containing processes. The ↑ DEGs in the sensitive genotype T following 24 h salt stress and compared to its respective control were highly representative of regulation processes such as protein phosphorylation, cellular protein modification, and establishment of proteins. The ↓ DEGs were carbohydrate metabolic, tetrapyrrole biosynthesis, and porphyrin−containing processes.

Comparing both analyzed genotypes under control conditions (T 0 vs. B 0) revealed that many more biological processes were enriched, including, response to stress, defense response and arginine catabolic process. Examination after 24 h salt stress (T 24 vs. B 24) processes specific to stress response such as to water and other abiotic stress and oxygen-containing compounds were allocated to the ↓ DEGs. The ↑ DEGs were enriched for protein phosphorylation and reproductive processes.

### 2.6. Expression of Salt-Stress Responsive Genes

Annotation of the identified DEGs in barley genotypes B and T after 24 h salt treatment revealed the involvement of several stress responsive genes. These included genes encoding signaling activities and proteins involved in osmoprotection and photosynthesis. Genes encoding functions such as reactive oxygen species (ROS) production and scavenging, and transcription factors were also indicated (Table S2).

Several signaling and regulation genes were differentially expressed in both barley genotypes after 24 h salt treatment. These genes included hormone-related genes, kinases, calcium sensors and phospholipases. Compared with the B genotype, more differentially expressed signaling genes were detected in T (Table S2).

Several components of diverse hormone-related pathways exhibited varying expression patterns under salt stress conditions. Among them, ABA signaling pathway ABC transporter C family members and the 2C-type protein phosphatases (PP2C) were the most abundant. Additionally, expression of several auxin-responsive proteins and SAUR-like auxin-responsive proteins was largely ↓ under salt stress. Furthermore, several ethylene and Giberellin signaling pathways were differentially regulated in the barley genotypes (Table S2).

Regarding protein kinases, the receptor-like kinase (RLK) gene and the Leucine-rich receptor-like kinase (LRR-RLK) gene family were mainly ↑ in T. However, most of the other kinase groups including serine-threonine kinases and protein kinases were ↓ particularly in T. In addition, Ca^2+^ signaling and phospholipase pathways were differentially regulated in response to salt stress (Table S2).

Following salt treatment, various components of photosynthesis were repressed in both barley genotypes, whereas their expression levels decreased more strongly in T than in B. The expression level of many photosynthetic genes was ↓ only in T, such as the those encoding photosystem II (PSII) reaction center PsbP family protein PPD4, the photosystem I reaction center subunit II and the PSII subunit X. Furthermore, plastocyanin and Rubisco genes were repressed only in T. Likewise, several chlorophyll a-b binding proteins and photoreceptors exhibited downregulation mainly in T compared to B (Table S2).

In addition, genes involved in cell wall structure including several cellulose synthase family proteins, xyloglucan endotransglucosylase-hydrolase, and genes involved in cell wall extension and degradation were repressed under salt stress mainly in the T genotype (Table S2).

In response to salt stress, several genes involved in major compatible solutes biosynthesis were differentially expressed in both barley genotypes, including proline, sugars and glycine betaine. Among sugars, activities related to glucose and trehalose metabolism were ↓ mainly in T. In contrast, sucrose synthases were ↑ particularly in B. Different types of sugar transporters were also differentially regulated (Table S2).

Genes involved in the generation of ROS, i.e., respiratory burst oxidase homologs (RBOH), were ↑ in both barley genotypes under salt stress. Simultaneously, enzymatic and non-enzymatic ROS scavenging systems were differentially expressed under salt treatment. Several APX and glutathione S–transferase (GST) genes were differentially expressed in both barley genotypes. Only one and two catalase (CAT) genes were ↑ in T and B, respectively, and only one superoxide dismutase (SOD) was ↓ in T. Likewise, several non-enzymatic thioredoxin and chalcone synthase families were ↓ in both genotypes, while only one tocopherol cyclase and one ferritin were ↓ particularly in T. The oxidative stress 3 (OXS3) gene was ↑ in B and ↓ in T (Table S2).

Components of various ion channel families and membrane transporters were differentially regulated in both the T and B genotypes whereas, salt overly sensitive Na^+^/H^+^ exchanger (SOS) was ↑ only in B (Table S2).

Expression of various transcription factor (TF) family members were differentially expressed in both genotypes. These included ethylene-responsive TF, a member of the APETALA2/ERF family, and basic helix-loop-helix DNA-binding (bHLH) superfamily members. Members of the Homeobox-leucine zipper, NAC, MADS-box, and myb-domain protein families were represented among DEGs along with ABA-responsive, protein-related and heat shock TFs. Some TF families such as WRKY and bZIP were differentially regulated only in T. Moreover, one member of the growth-regulating factors family, GRF4, was repressed only in T (Table S2).

### 2.7. Co-Expressed DEGs in Response to Salt Stress

**Figure 6:**
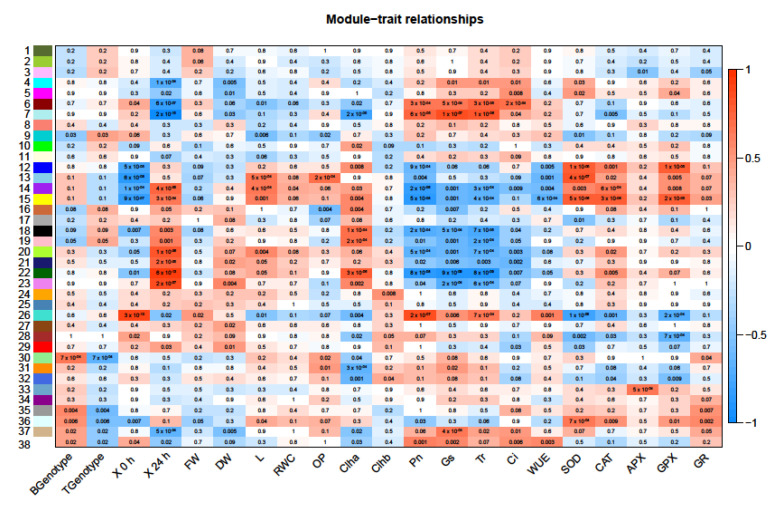
Correlation of WGCNA co-expression modules to each genotype. The analysis considered several parameters including genotype (Boulifa, B and Testour, T), stress duration (0 h, 24 h) and morpho-physiological traits (fresh weight (FW), dry weight (DW), shoot length (L), relative water content (RWC), osmotic potential (OP), chlorophyll a/b content (Chl and Chl b), net CO_2_ assimilation rate (Pn), stomatal conductance (Gs), transpiration rate (E), internal concentration of CO_2_ (Ci), water use efficiency (WUE), superoxide dismutase (SOD), catalase (CAT), ascorbate peroxidase (APX), guaiacol peroxidase (GPX), and glutathione reductase (GR)). Each numbered row corresponds to a module, each column to a trait (labelled along *x*-axis). Each cell contains the corresponding *p*-value and is color-coded by the strength of correlation according to the legend on the right (red and blue shading indicates level of correlation and ranges from +1 to −1).

Weighted gene co-expression network analysis (WGCNA) was used to identify modules (clusters) of co-regulated genes that correlated with the morpho-physiological traits and genotypes. A total of 38 modules were identified and are numbered and color-coded on the *y*-axis of Figure 6.

Modules where expression levels were correlated with genotypes and morpho-physiological traits, namely modules 38, 36, 26, 22, 18, and 15, were further characterized. GO enrichment analysis indicated that several pathways were enriched for these modules (Supplementary Figure S1). Module 38 was positively correlated with the B genotype as well as all photosynthetic traits (Pn, Gs, Tr, Ci, and WUE). Module 36 correlated positively with B genotype and antioxidant enzymes (SOD, CAT, APX, GPX, and GR).

Module 26 was positively correlated with the T genotype and control conditions as well as growth traits (FW, DW), Chl b, and all photosynthetic traits but was negatively correlated with all antioxidant enzyme activities. Module 22 was positively correlated with the T genotype, growth traits (DW, L, and RWC), Chl a, and antioxidant enzymes (SOD, CAT, APX, GPX, and GR) but was highly negatively correlated with all photosynthetic traits (Pn, Gs, Tr, Ci, and WUE). Module 18 was positively correlated with T genotype and salt treatment as well as growth traits, Chl a, and antioxidant enzymes and negatively correlated with all photosynthetic traits. Module 15 was positively correlated with B genotype, salt treatment and growth traits (DW, L), Chl a, and all antioxidant enzymes but negatively correlated with all photosynthetic traits.

With respect to molecular function, GO analysis indicated that the most enriched categories in all modules were protein binding, transferase, kinase, and oxidoreductase activities. In module 38, transporter activity (ion/cation channel) was also enriched (Figure S2).

Regarding biological process, the most enriched categories were highly similar in all modules (metabolic, cellular and biological regulation processes, and localization), and the predominant annotated sub-categories varied depending on modules (Table 4; Figure S2). For instance, module 38 positively correlated with the tolerant genotype B, 0 h salt stress as well as all photosynthetic activities, and was also enriched in lipid biosynthetic process and transporter activity. In addition, module 36, which positively correlated with the tolerant genotype B, 24 h salt stress as well as all antioxidant enzyme activities, was also enriched for protein acetylation. Modules 18 and 26, positively correlated with the sensitive genotype T, were enriched in primary metabolic process and protein phosphorylation.

**Table 4:** The predominant annotated biological process categories and subcategories in the six selected modules.

Module	GO Category	GO Subcategory
15	metabolic process	RNA metabolic process
	cellular process	organelle organization
	localization	intracellular transport
38	metabolic process	lipid biosynthetic process
	cellular process	microtubule−based process
36	metabolic process	primary metabolic process
	metabolic process	protein acetylation
	metabolic process	histone acetylation
	cellular process	transcription by RNA
	cellular process	regulation of transcription
	biological regulation	organelle organization
	biological regulation	chromosome organization
22	metabolic process	RNA metabolic process
	metabolic process	transcription by RNA
	cellular process	organic cyclic compo…
	cellular process	nucleobase−containing
	biological regulation	regulation of protein
	biological regulation	regulation of catabolism
	biological regulation	ion homeostasis
	biological regulation	regulation of pH
26	metabolic process	primary metabolic process
	metabolic process	macromolecule modification
	cellular process	phosphate−containing
	cellular process	protein phosphorylation
18	metabolic process	primary metabolic process
	metabolic process	macromolecule modification
	cellular process	phosphate−containing
	cellular process	protein phosphorylation

### 2.8. RNA-seq Data Validation by RT-qPCR

**Figure 7:**
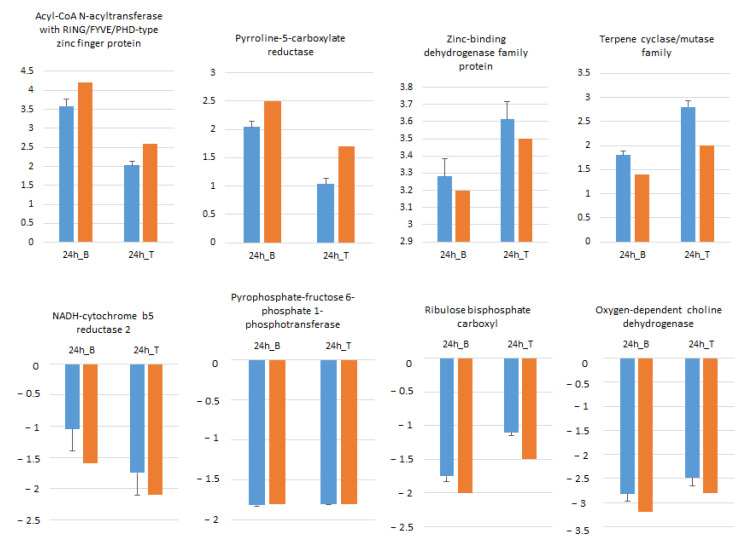
Expression pattern validation of eight randomly selected genes in Boulifa and Testour leaves by qRT-PCR. The blue bar indicates the relative expression level determined by qRT-PCR using the 2^−ΔΔCT^ method mean with associated standard error bar (*n* = 3) and alpha tubulin (TUB2) was used as reference gene for data normalization. The orange bar represents transcript abundance changes of RNA-seq data calculated by the Log2 fold change method.

Eight randomly selected genes including four ↑ and four ↓ genes in B and T genotypes were analyzed by qRT-PCR in order to confirm RNA-seq data. As illustrated in Figure 7, the expression profiles of all selected genes at 24 h salt treatment compared to control conditions were in accordance with RNA-seq data.

## 3. Discussion

In this study, an integrative approach was employed to explore the role of gene expression in the differences in salt stress response between barley cultivars Boulifa (salt-tolerant) and Testour (salt-sensitive). We exploited the differential salt-tolerance response of these two genotypes to assign DEGs to various functional and biological processes. Additionally, WGCNA was used to elucidate specific gene modules differing between the two barley genotypes and subsequent correlation of modules with morpho-physiological traits.

When compared to their respective controls, the T phenotype was affected more by salt stress than the B phenotype. Growth characteristics were impacted including shoot length and fresh and dry leaf biomass. Compared to control plants the dry weight decrease was three times more pronounced in T than B. Similar repercussions of salt stress have been reported previously for barley [28] and maize [20]. Growth repression likely resulted from the effect of osmotic salt stress reducing water uptake [36,37]. Following 24 h salt treatment a greater decrease in RWC was also detected in T compared to B relative to their respective control plants, suggesting better water retention in B [37]. In addition to reduced water uptake and retention, decreases in several parameters associated with photosynthesis [28,29] and induced oxidative stress [38,39] may have contributed to the observed repression in plant growth. Consistent with previous reports [28,40], the sensitive genotype T exhibited stronger inhibition of all photosynthetic parameters (Pn, Gs, E, Ci and WUE) including photosynthetic pigment synthesis (Chl a and Chl b).

Stomatal conductance (Gs) was used to screen for salt tolerance in durum wheat by Rahnama et al. [41]. Here, Gs was reduced more than 2.5-fold in T compared to B relative to their respective controls. Tolerant barley plants maintained Gs rates under saline conditions, preventing water loss and promoting better yield [28]. Degradation of Chl b in the sensitive genotype T was greater than B in response to salt stress relative to their respective controls, corroborating previous studies [29,40], and could be due to higher induced ROS damage [29]. More greatly increased activity of antioxidant enzymes in B, mainly SOD, CAT and GPX, emphasized the role of induced ROS protection in improved salt tolerance [32,40]. These results suggest that the more robust maintenance of physiological salt tolerance mechanisms in B improved its capacity to maintain a relatively higher growth rate under salt stress.

The phenotypic differences in salt tolerance between B and T genotypes were supported by the RNA-seq analysis that revealed a much greater number of DEGs in T than B after 24 h salt treatment when compared to their respective controls. Approximately 2.6 times more salt-responsive genes were differentially regulated in T than in B. This suggests that the transcriptional response of the sensitive genotype T was strongly altered by the salt stress exposure. Low DEG abundance in tolerant genotypes relative to sensitive genotypes exposed to salt stress has been demonstrated for several plant species [42,43]. Ruiz et al. [43], reported fewer DEGs in glycophytes compared to halophytes under salt stress, suggesting that increases in DEGs under salt stress could be considered an indicator of plant sensitivity [44].

In the sensitive genotype T, 78.2% of DEGs were overexpressed and 21.8% were repressed under salt stress. Although there were fewer DEGs detected in the tolerant B genotype, similar ↑ and ↓ DEG proportions were detected. Several previous studies reported similar trends of DEG distribution between salt-sensitive and –tolerant genotypes of different species including barley [21], alfalfa [42], and quinoa [43]. The current results emphasize that the differential regulation of gene expression in T and B barley genotypes likely underlie the differential morpho-physiological response to salinity at an early stage of development.

### 3.1. Gene Expression Related to Photosynthesis, Osmoregulation, Oxidative Stress Response and Ion Homeostasis

Photosynthesis is the main source of energy needed for plant metabolism. It has been widely demonstrated that salt stress reduces photosynthetic efficiency, thus inhibiting plant growth via reduction of available resources and decreased cell division and expansion [28,29,45,46]. RNA-seq analyses revealed downregulation of several genes encoding major components of the photosynthetic reaction centers PSI and PSII. The core reactions of photosynthesis include NADP^+^ reduction and water splitting at PSI and light absorption at PSII. Both reaction centers participate in responses to environmental stress conditions [47,48]. In the sensitive T genotype 10 PSII components were repressed however only four components were ↓ in the tolerant B. Likewise, five PSI subunits were repressed in T compared to only one in B. Similar results were noted for barley leaves subjected to drought stress where repression of components in both photosystems was low in the drought-tolerant genotype compared to the drought-sensitive genotype [25]. Notably, plastocyanin, an electron transporter associated with photosynthesis, and Rubisco (ribulose-1,5-bisphophate carboxylase/oxygenase) accumulation factor 1, were ↓ only in T. Furthermore, the light receptors chlorophylls a and b and the chlorophyll a-b binding protein that capture and deliver excitation energy to PSI and PSII were highly ↓ in T compared to B. The differential regulation of photosynthesis-related genes in both genotypes promoted a more protective stomatal and non-stomatal response in B as exhibited by the smaller decline of all photosynthetic parameters (Pn, Gs, E, Ci and WUE). The greater accumulation of chlorophyll a and b in B relative to T suggests that stomatal and non-stomatal inhibition of chlorophyll synthesis could contribute to salinity tolerance in barley plants [29]. Marginal reduction in RWC in B relative to T may also point to stomatal response permitting growth even under saline conditions [28].

The higher photosynthetic efficiency of B could be related to cell wall organization in response to salinity. Downregulation of several genes involved in the biosynthesis of the cell wall such as the cellulose synthase protein, xyloglucan endotransglucosylase /hydrolase (XTH), was greater in T compared to B. Recognized as a cell-wall-modifying enzyme, XTH is involved in diverse physiological processes and its overexpression in *Arabidopsis* transgenic plants improves salt tolerance [49]. This finding is in agreement with previous RNA-seq studies on barley, maize, and plum genotypes with contrasting drought tolerance characteristics [25,34,50]. Notably, the glycine-rich cell wall structural protein was ↑ only in B. This protein is involved in cell wall plasticity and its overexpression confers enhanced tolerance to diverse abiotic stresses including salinity [51]. In addition, the hydroxyproline-rich glycoproteins (HRGP), with major roles in cell wall signal transduction cascades, plant development and stress tolerance [52] were more repressed in T compared to B. Furthermore, the arabinogalactan protein, a member of the HRGP family, was shown to be ↑ in response to salt stress in salt-adapted tobacco [52].

Wound-induced cell wall protein 1 (WIN1) transcripts rapidly increased in response to mechanical wounding and may correlate with cell death as it accumulates in senescing tissues [53]. Expression of win1 was ↑ in T and ↓ in B. This may be responsible for the more robust growth of B shoots under salt treatment. According to Savatin et al. [54], the increased accumulation of win1 transcripts was inhibited by adding osmoprotectants, emphasizing the importance of osmotic adjustment to sustain plant growth under adverse conditions [32].

Osmotic adjustment is positively correlated with stress tolerance. To cope with osmotic stress, plants activate the biosynthesis of diverse compatible solutes [28]. The major compatible solutes, including proline, sugars, and glycine betaine, accumulate under salt stress and tend to maintain low intracellular osmotic pressure by adjusting cytoplasmic water content, preventing harmful effects of salt stress [55]. The current RNA-seq data revealed that several key genes involved in the biosynthesis of these osmoprotectants were differentially regulated by salt stress in both genotypes.

Pyrroline-5-carboxylate reductase, which catalyzes the terminal step in proline biosynthesis from glutamate [56], was induced in both genotypes under salt stress. However, its expression was elevated more in B compared to T. Osmoprotectant glycine betaine is involved in the plant salt stress response [57]. Oxygen-dependent choline dehydrogenase, an enzyme involved in glycine betaine synthesis, was similarly repressed in both genotypes. Betaine aldehyde dehydrogenase 2, which catalyzes the conversion of betaine aldehyde from betaine, was induced only in B [58].

The expression of sugar biosynthetic genes, e. g. sucrose synthases, was differentially regulated in both genotypes. Sucrose synthase 1 and 6 were ↓ and ↑ in both genotypes, respectively. However, sucrose synthase 4 was ↑ only in B. Similarly, Cui et al. [59] detected elevated sucrose synthase activity and relatively high amounts of sucrose in low-temperature-treated *Medicago*. The GO enrichment analysis revealed overrepresentation of the subcategories of sucrose metabolic and organic substance metabolic processes. This highlights the involvement of osmotic and photosynthetic homeostasis in preventing salt stress damage and sustaining an improved growth rate under stressful conditions in the tolerant genotype B.

Overall, the relatively higher upregulation of some genes encoding osmoprotectants in B genotype could explain its better photosynthetic activity and higher leaf water content. Osmolytes can preserve cell membrane stability, prevent oxidative damage, and even act as salt-stress signaling molecules that influence stress-related gene expression [57]. Several studies have reported increased activity of enzymatic and non-enzymatic antioxidant components in salt tolerant plants following the elevation of ROS after salt treatment [46,60].

Genotype-specific transcriptional changes were detected for several peroxidases including ascorbate peroxidase. Glutathione S–transferase, catalase, and superoxide dismutase also followed genotype-specific patterns. For instance, catalase 3 was ↑ only in B and Fe superoxide dismutase 2 was ↓ only in T. Differential expression regulation of antioxidant genes under salt stress may explain the enhanced activities of their encoded enzymes mainly in B genotype. These results corroborate the reports of Yousefirad et al. [16] on mutant and wild barley subjected to salt stress and Harb et al. [25] on drought stress response of contrasting barley genotypes. The overexpression of APX and GST genes enhances salt tolerance in transgenic *Arabidopsis* plants [61,62]. Moreover, the upregulation of the oxidative stress 3 (OXS3) gene, involved in tolerance to oxidative stress, in B and its downregulation in T could emphasize the better oxidative stress tolerance in the B genotype. Indeed, the *Arabidopsis* mutant and overexpression lines of OXS3 exhibited oxidative stress sensitivity and tolerance, respectively [63].

Non-enzymatic ROS scavenging genes, including the thioredoxin family, protect cell components from damage under adverse conditions [60]. Differential regulation of this gene category was more dramatic in T compared to B. In addition, expression of the tocopherol cyclase, an antioxidant metabolite related gene, involved in protecting lipids from oxidation under environmental stresses [60,64], was exclusively ↓ in T. This highlights the greater need to protect against oxidative stress in the T genotype.

Several ion transporters were also differentially expressed in barley leaves in response to salt stress. Under salt stress, a high cytosolic K^+^/Na^+^ ratio is crucial for plants to survive [5]. The sodium transporter HKT is essential in maintenance of ion homeostasis under salinity conditions, a crucial role in plant salt tolerance [65,66]. Expression of this transporter was similarly enhanced in both barley genotypes following 24 h salt exposure. Potassium transporters, which influence salt tolerance through involvement in regulation of K^+^ absorption in leaves [65], were likewise upregulated in both genotypes. Upregulation of these ion transporters in response to saline conditions concurs with previous transcriptomic reports on mutant barley [16] and maize [20]. In contrast, expression of SOS1, involved in Na^+^ transport [67], was enhanced only in B. Overexpression of SOS1 significantly enhanced salt tolerance in *Arabidopsis* transgenic lines [68] suggesting that its upregulation in B influenced the salt tolerance of this genotype.

### 3.2. Signaling and Regulatory Proteins

Effective plant response to harmful conditions requires stress perception and signal transduction to activate expression of target genes. Several kinase signaling gene families were differentially expressed in both barley genotypes following salt stress treatment. Several MAPK kinases were overrepresented among the downregulated genes, especially in T. Members of the leucine-rich receptor-like protein kinase family (LRR-RLK), including the LRR receptor-like serine/threonine-protein kinase EFR (elongation factor Tu receptor) was downregulated. These family members are involved in both biotic and abiotic stress responses [69] and overexpression of LRR-RLK in *Arabidopsis* enhanced WUE [70]. The downregulation of several kinases in T compared to B could be one of the factors associated with salt sensitivity. These results concur with previous studies on barley and plum cultivars with contrasting drought stress responses [25,50].

### 3.3. Transcription Factors

Transcription factors, regulators of stress-related genes, are distributed in several gene families such as MYB, bZIP, NAC, CBF/DREB, HSF, WRKY, and ABF/ABRE [5]. According to our RNA-seq data, salt stress highly influenced expression of diverse TF gene families, especially in T.

The WRKY gene family is one of the largest plant TF families with members involved in plant development and stress responses [71]. One of the most important functions of WRKY genes is regulation of the salt stress response. Overexpression of several WRKY genes from maize and wild cotton (*Gossypium aridum*) enhanced the salt tolerance of transgenic *Arabidopsis* plants compared to wild type [72,73]. WRKY genes were differentially regulated exclusively in salt-stressed T. Three genes were ↑ and one ↓, emphasizing the complexity of transcriptional regulation and the involvement of several factors to overcome salinity [74].

The MYB domain-containing genes encode TFs widely involved in abiotic stress response, growth and development in plants [75]. These TFs have been associated with ROS signaling and induction of oxidative stress responses [74] and their overexpression in rice transgenic plants improved tolerance to freezing, dehydration and salt stress [76]. Several MYB domain genes were differentially regulated in both barley genotypes under salt treatment. In B, transcription of three MYB genes was enhanced, while transcription of three others was repressed. In T, expression of one MYB was enhanced while expression of four was repressed. The three induced MYB genes in B, *MYB63*, *MYB20*, and *MYB41*, are activators of lignin and wax biosynthesis in *Arabidopsis* and their disruption resulted in developmental defects [77,78]. Furthermore, *MYB20* and *MYB41*, positively regulate ABA signaling in response to salt stress, and transgenic *Arabidopsis* lines overexpressing these two MYB genes showed enhanced salt tolerance [79]. Expression of *MYB63* was highly ↑ in B relative to T; however *MYB20* and *MYB41* were induced only in B. The higher induction of these three MYB genes in B may be important for salt tolerance in this genotype.

Another group of TFs belong to the bHLH gene family and are efficient regulators of biosynthesis of several secondary metabolites, inducing flavonoids and anthocyanins involved in stress responses [74,80]. Overexpression of several bHLH genes increased the tolerance of different transgenic plants against drought and salinity by increasing flavonoids and anthocyanin accumulation [81,82]. In this study, three bHLH TFs were similarly regulated in both barley genotypes, two were enhanced and one was repressed.

It is worth noting that growth-regulating factor 4 (GRF4), a plant-specific TF with roles in stem and leaf cell expansion, proliferation, and development, and associated with growth maintenance under adverse environmental conditions was ↓ only in T. Overexpression of various GRF genes in *Arabidopsis* leads to larger leaf size compared to wild type [83]. Moreover, Huang et al. [84] found elevated *GRF4* gene expression in wheat leaves treated with 96 h NaCl compared to control plants. This gene may play an active role in plant response to salt stress.

Overall, more regulatory processes were detected in the sensitive genotype T compared to B, implying that a more complicated physiological process occurred in T than in B when exposed to salt stress.

### 3.4. Identification of Genetic Modules Corresponding to Salt Stress

Weighted correlation network analysis (WGCNA) is an efficient tool for data exploration allowing gene screening related to traits and classification of co-expressed clusters with high biological significance [85]. We identified 38 gene modules, among them, six modules highly correlate transcript levels with genotypes and morpho-physiological traits (Figure 6).

Annotation of these modules confirmed that salt stress response mechanisms were genotype specific. Indeed, the modules (15, 36, 38), which correlated significantly with B genotype, also showed positive significant correlations with either photosynthetic or oxidative traits, which was not the case for the modules that correlated with T genotype. These data corroborate our findings from the physiological assays and highlight the possible use of physiological and/or antioxidant enzymes as salt stress tolerance markers.

Furthermore, GO enrichment analysis of the modules that significantly correlated with the tolerant genotype B revealed that lipid biosynthetic, protein acetylation (affecting diverse aspects of protein function such as enzymatic stability and activity [86]), and microtubule (function in the plant salt stress response [87]) processes were associated with these modules. Elevation of expression for genes involved in these processes could explain the higher growth maintenance in B genotype after 24 h salt treatment. The white module (#38) enriched for the GO annotation terms lipid biosynthesis and microtubule process, was significantly correlated with B at 0 h salt stress (absence of salt). In addition, several biological processes were enriched under control conditions when comparing T 0 vs. B 0, including response to stress and defense response. This could explain the fewer number of DEGs in B under 24 h salt stress compared to T and suggests that the salt tolerance of B is genotype-specific emphasizing the importance of the genotype itself to overcome stress [88,89].

The modules (18, 22, 26) that significantly correlate with the sensitive genotype T were enriched in several regulation processes, including that of protein, catabolism, primary metabolic processes, and protein phosphorylation, key steps in almost all cellular activity [90]. This is in accordance with more complex signaling and transcriptional regulation detected in the T genotype since more energy is needed for the sensitive genotype to overcome stress.

## 4. Conclusions

In this study, two barley genotypes differing in their tolerance to salinity (Boulifa and Testour) were evaluated for differential gene expression, antioxidant enzyme activity, and physiological responses following 200 mM NaCl treatment. Relative to Testour, the tolerant genotype Boulifa had better photosynthetic capacity, higher expression of antioxidant enzymes and activated expression of relevant biosynthetic pathways more strongly. This allowed better osmotic protection and antioxidant response, conferring differential growth performance between tested genotypes that was supported by higher, and possibly maladaptive, levels of transcriptional regulation in Testour compared to the Boulifa. In addition, comparison of leaf transcriptomes between salt-tolerant and sensitive genotypes following salt exposure allowed the identification of key candidate genes enhanced exclusively in the tolerant B including sucrose synthase 4, catalase 3, OXS3, and SOS1 as well as several TFs such as MYB20 and MYB41 and GRF4. Future studies should consider delving more deeply into the relationship between these factors and salt-stress responses to inform barley-breeding programs aimed at increasing salinity tolerance.

## 5. Materials and Methods

### 5.1. Plant Material, Growth Conditions, Salt Stress Treatment and Physiological Measurements

Seeds of two genetically distinct Tunisian barley landraces Boulifa (B, salt-tolerant) [91] and Testour (T, salt-sensitive) [35,92] were germinated in Petri dishes. Five-day-old seedlings were transferred to an aerated hydroponic system under controlled conditions (photoperiod: 16 h light/8 h dark, temperature: 25/19 °C, and relative humidity: 65%), acclimated for 3 days, and subjected to gradual salt stress application up to 200 mM following established methods [91]. Severe salt stress (200 mM) was adopted to allow clear discrimination between sensitive and tolerant genotypes without being drastic [35]. Leaves were harvested at 0 h (before adding NaCl), then again at 2, 8, and 24 h after exposure to 200 mM NaCl. At each time point, three pools of five plants of both genotypes were considered for antioxidant enzyme assays and RNA-seq analysis. Pooled samples were frozen in liquid nitrogen and stored at −80 °C. Growth assessment was conducted at 24 h after salt treatment on control and stressed leaves by measuring length (L) and fresh and dry weights (FW and DW, respectively). Relative water content (RWC) was calculated as RWC (%) = [(Fresh Weight − Dry Weight)/(Turgid Weight − Dry Weight)] × 100 [93] and osmotic potential (OP) was measured on leaf extracts using an osmometer (osmomat 3000 Type D-10553, Berlin, Germany) [26].

Photosynthetic parameters, namely net CO_2_ assimilation rate (A), stomatal conductance (gs), transpiration rate (E), and internal concentration of CO_2_ (Ci) were also measured on control and stressed plants after 24 h of 200 mM NaCl treatment. Measurements were done under atmospheric CO_2_ using a portable photosynthetic system (LCpro^+^, Inc., Hoddesdon, UK) on the last fully expanded leaf. The data were collected at 10:00 am at an ambient CO_2_ concentration of 360 μmol mol^−1^ and photosynthetic active radiation in the leaf chamber of 980 µmol m^−2^ s^−1^. At the same time, point, chlorophyll a (Chl a) and chlorophyll b (Chl b) contents were spectrophotometrically determined according to Lichtenthaler [94].

### 5.2. Antioxidant Enzymes Assays

The extraction of antioxidant enzymes was performed from 500 mg of frozen leaf samples using 50 mM phosphate buffer solution (pH 7.8) added with 10% polyvinylpyrrolidone (PVP), 1 mM phenylmethylsulfonyl fluoride (PMSF), 2 mM EDTA, 10 mM DTT, and 0.1% triton X-100. For APX activity measurement, 5 mM ascorbate was added to the extraction buffer. The homogenates were centrifuged at 13,800 rpm for 15 min at 4 °C and supernatants were collected [95]. Protein contents (μg μL^−1^) were determined at 595 nm using a spectrophotometer following the method of Bradford [96]. SOD activity was evaluated by measuring the photoreduction inhibition of nitroblue tetrazolium at 560 nm [97]. CAT activity was detected by monitoring the degradation rate of H_2_O_2_ at 240 nm according to Cakmak and Marschner [98]. APX activity was assayed by following the rate of H_2_O_2_-dependent ascorbate peroxidation at 290 nm [99]. GPX activity was assessed by recording the increase in absorbance at 470 nm due to the guaiacol oxidation [100]. GR activity was measured following the GSSG (oxidized glutathione)-dependent oxidation of NADPH by the decrease in absorbance at 340 nm [101].

### 5.3. RNA Isolation, DNase Treatment and Sequencing

Three pools of leaves, each of which contained five control or stressed plants of the two studied genotypes, were used for total RNA isolation. The sampling was done at 0, 2, 8, and 24 h after salt stress application [91]. RNA extraction was performed using the ZR Plant RNA MiniPrep™ Kit (Zymo Research, Irvine, CA, USA). DNA was eliminated from RNA samples by TURBO DNA-free™ Kit (Promega, Madison, WI, USA). The quality and quantity of the isolated RNAs were checked by agarose gel electrophoresis (1%) and spectrophotometrically using BioPhotometer (Eppendorf BioPhotometer plus, Hamburg, Germany).

The three replicates of each treatment for both genotypes were sequenced at the Beijing Genomics Institute (BGI, Shenzhen, China) using the Illumina NextSeq 500 platform as described in our previous publication [91].

The clean sequencing reads were submitted to SRA database at NCBI (accession number: PRJNA821484).

### 5.4. Pseudoalignment and Differential Expression Analyses

The reads were pseudo-aligned to the barley transcriptome (from pigs barley genome database 2017) using Kallisto and transcript-level abundances were obtained. These were aggregated to gene-level abundances using tximport and normalized using DESeq2. Principal component analysis and hierarchical clustering was performed using 500 genes with the highest variance in expression in order to explore the underlying structure of the data in an unsupervised manner. Differential expression analysis was performed separately for the four contrasts: T 0 vs. B 0, T 24 vs. B 24, B 24 vs. B 0, and T 24 vs. T 0 in order to understand the relationship between salt stress and the two genotypes. DeSeq2 was used to normalize the gene abundances, estimate dispersion, model the abundances as a negative binomial distribution and perform a Wald test to identify significantly differentially expressed genes. Genes with adjusted *p*-value (after Benjamini–Hochberg correction) ≤0.01 and absolute log 2-fold change ≥1 were reported as significantly differentially expressed in each contrast.

### 5.5. Functional Enrichment Analysis of DEGs

Gene ontology terms enriched among each set of differentially expressed genes were identified using topGO, an R package for GO enrichment analysis. The differentially expressed genes for each contrast were separated into upregulated genes (adjusted *p*-value ≤ 0.01 and log 2-fold change ≥1) and downregulated genes (adjusted *p*-value ≤ 0.01 and log 2-fold change ≤ −1). A classic fisher enrichment test was performed using each list of differentially expressed genes to identify GO terms that were significantly overrepresented among that list. GO terms in the molecular function and biological process domains were identified in order to identify the biological functions likely dysregulated based on the differentially expressed genes.

### 5.6. Weighted Gene Co-Expression Network Analysis (WGCNA)

The WGCNA R package was used to construct a scale-free co-expression network using all the genes in the dataset. WGCNA, rather than relying on *p*-value and fold change cutoffs to choose genes of interest, uses all the genes in the dataset to identify clusters of co-expressed genes called modules. Using correlation as a measure for co-expression, genes were clustered based on similarity in expression and a dynamic tree-cutting algorithm was used to identify modules (minimum size of 30 genes). The eigengene (most representative gene) of each module was correlated with metadata such as genotype, time point and physiological traits to identify modules of interest. Genes belonging to these modules were extracted and topGO was used to associate functional terms with each module of interest.

### 5.7. Quantitative Real-Time PCR Analysis

DNase treated RNAs were used for cDNA synthesis according to the manufacturer’s instructions of the GoScript™ Reverse Transcription System Kit (Promega). The qRT-PCR reactions were performed using the 7300 Real-Time PCR Detection System (Applied Biosystems, Foster City, CA, USA) and the Applied Biosystems Power SYBR Green qPCR Master Mix (Life technologies, Carlsbad, CA, USA). The 2^−∆∆CT^ method of Schmittgen and Livak [102] was used to calculate the relative expression levels of the target genes and alpha tubulin (TUB2) was used as reference gene for data normalization. Fold change was calculated for the salt-treated plants relative to the controls. The primers used were designed by the Primer3 Input (version 0.4.0) software (created by Steve Lincoln, Mark Daly, and Eric S. Lander; http://bioinfo.ut.ee/primer3-0.4.0/) accessed on 22 November 2021 [103] and are listed in the Supplementary Table S3.

### 5.8. Statistical Analysis

The morpho-physiological data were analyzed by one-way analysis of variance (ANOVA). All experiments were repeated three times independently with six replications per treatment and the values are presented as mean ± standard error (SE). Means were separated by Tukey’s post hoc test (*p* ≤ 0.05) using SPSS program (SPSS software, version 11/PC SPSS 11.0, Inc., Chicago, IL, USA, 2001) developed by (Norman H. Nie, C. Hadlai (Tex) Hull and Dale H. Bent).

## Figures and Tables

**Table 3:** Antioxidant enzyme activities in leaves of control and salt-stressed seedlings.

Genotype	Treatment	SOD	CAT	APX	GPX	GR
Boulifa	Control	0.77 ± 0.04 ^c^	0.79 ± 0.08 ^c^	1.17 ± 0.10 ^a^	1.40 ± 0.07 ^c^	0.61 ± 0.02 ^a,b^
	Salt stress	1.54 ± 0.08 ^a^	1.87 ± 0.04 ^a^	1.13 ± 0.04 ^a^	3.80 ± 0.15 ^a^	0.67 ± 0.04 ^a^
Testour	Control	0.79 ± 0.02 ^c^	0.74 ± 0.03 ^c^	0.90 ± 0.02 ^a,b^	1.45 ± 0.08 ^c^	0.58 ± 0.06 ^b^
	Salt stress	1.00 ± 0.08 ^b^	1.10 ± 0.05 ^b^	1.11 ± 0.11 ^a^	2.15 ± 0.09 ^b^	0.60 ± 0.07 ^a,b^

Data are mean ± SE of three replicates. Different lower-case letters (a, b, c) indicate that means in the same column with different superscripts are significantly different (Tukey’s HSD, *p* ≤ 0.05). Superoxide dismutase (SOD), catalase (CAT), ascorbate peroxidase (APX), guaiacol peroxidase (GPX), and glutathione reductase (GR). All enzyme activities are reported in mM mg^−1^ min^−1^ protein.

## Data Availability

The data presented in this study were submitted to the NCBI Sequence Read Archive (accession number: PRJNA821484).

## Supplementary Materials

The following supporting information can be downloaded at: https://www.mdpi.com/article/10.3390/ijms23095006/s1.
