# Supplementary material for: Effects of Salt Stress on Transcriptional and Physiological Responses in Barley Leaves with Contrasting Salt Tolerance
Source: Int J Mol Sci. 2022 Apr 30;23(9):5006. doi: 10.3390/ijms23095006 (PMC9103072; doi:10.3390/ijms23095006)
Supplement: Supplementary file 1 [file ijms-23-05006-s001.zip › Supplementary Figures.pptx]

## Slide 1
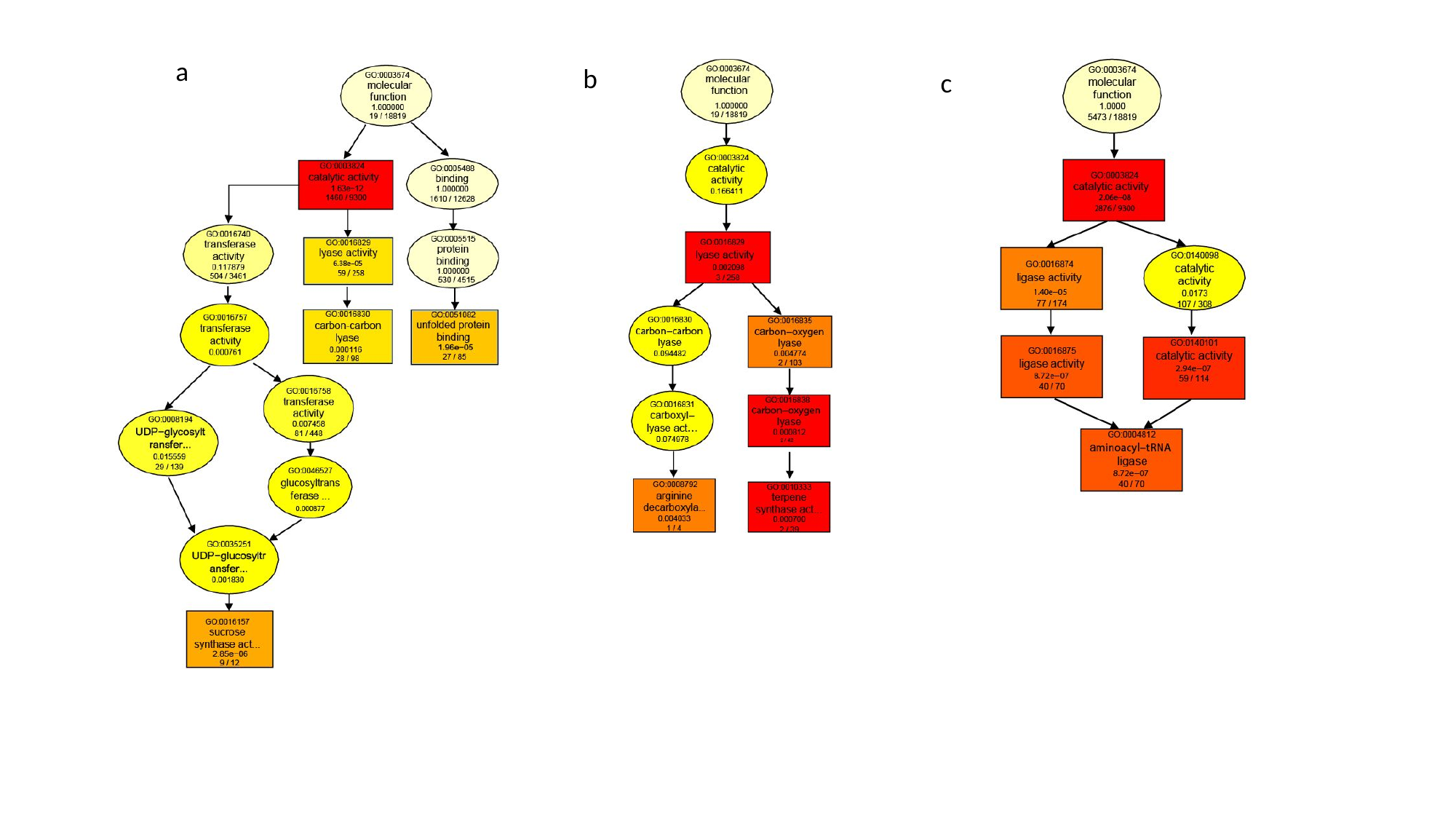

a
b
c

## Slide 2
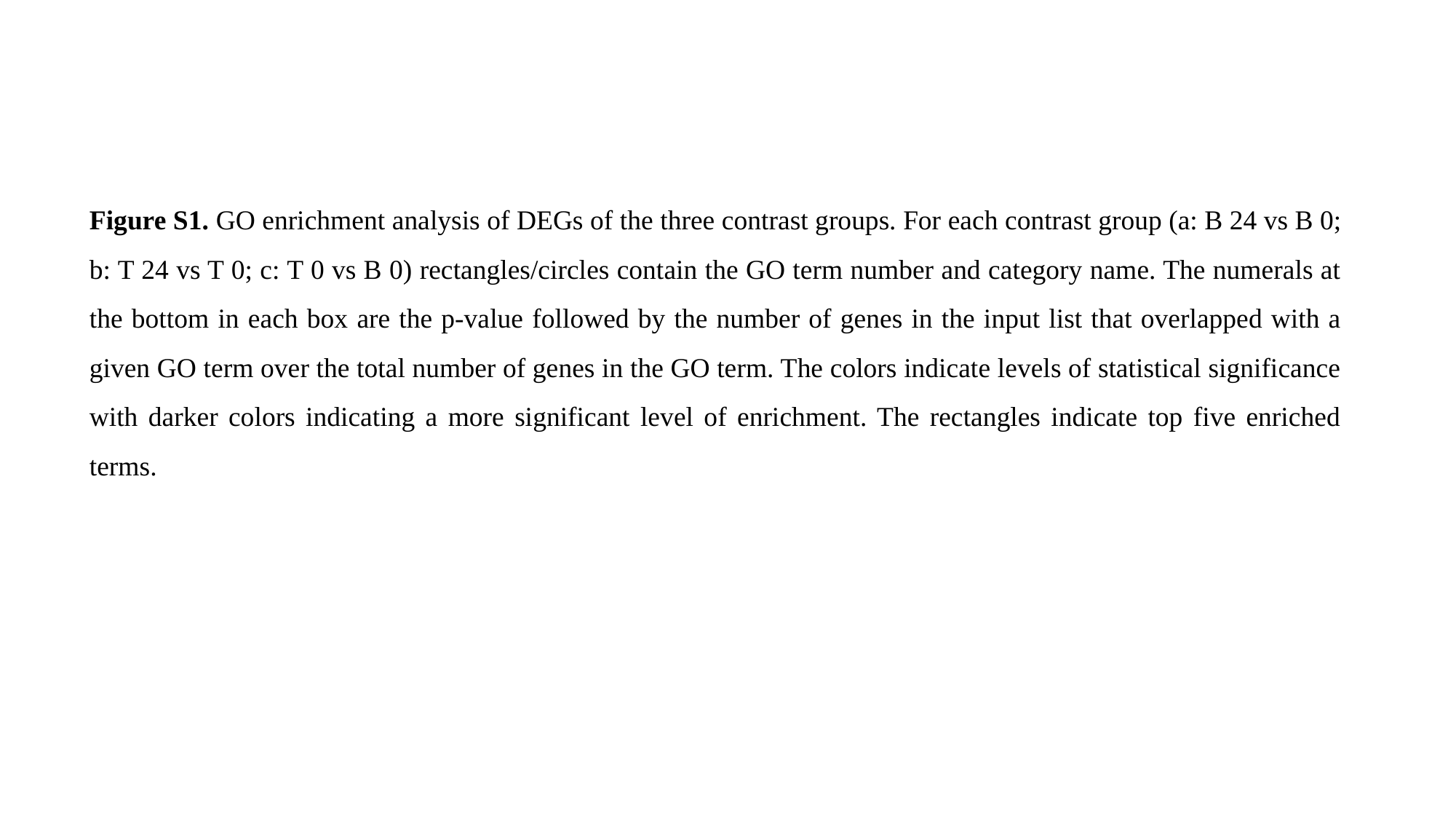

Figure S1. GO enrichment analysis of DEGs of the three contrast groups. For each contrast group (a: B 24 vs B 0; b: T 24 vs T 0; c: T 0 vs B 0) rectangles/circles contain the GO term number and category name. The numerals at the bottom in each box are the p-value followed by the number of genes in the input list that overlapped with a given GO term over the total number of genes in the GO term. The colors indicate levels of statistical significance with darker colors indicating a more significant level of enrichment. The rectangles indicate top five enriched terms.

## Slide 3
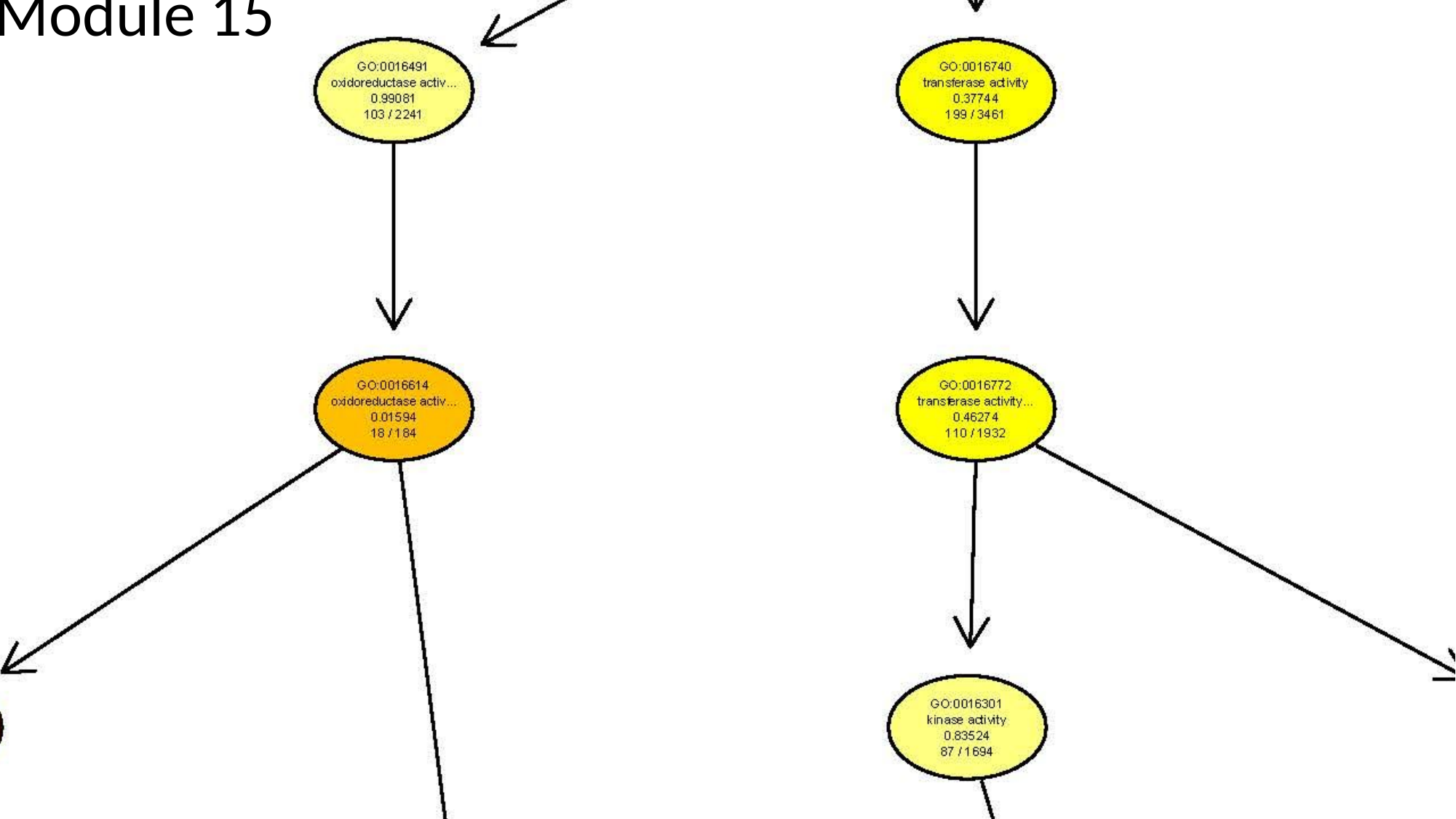

MF: Module 15

## Slide 4
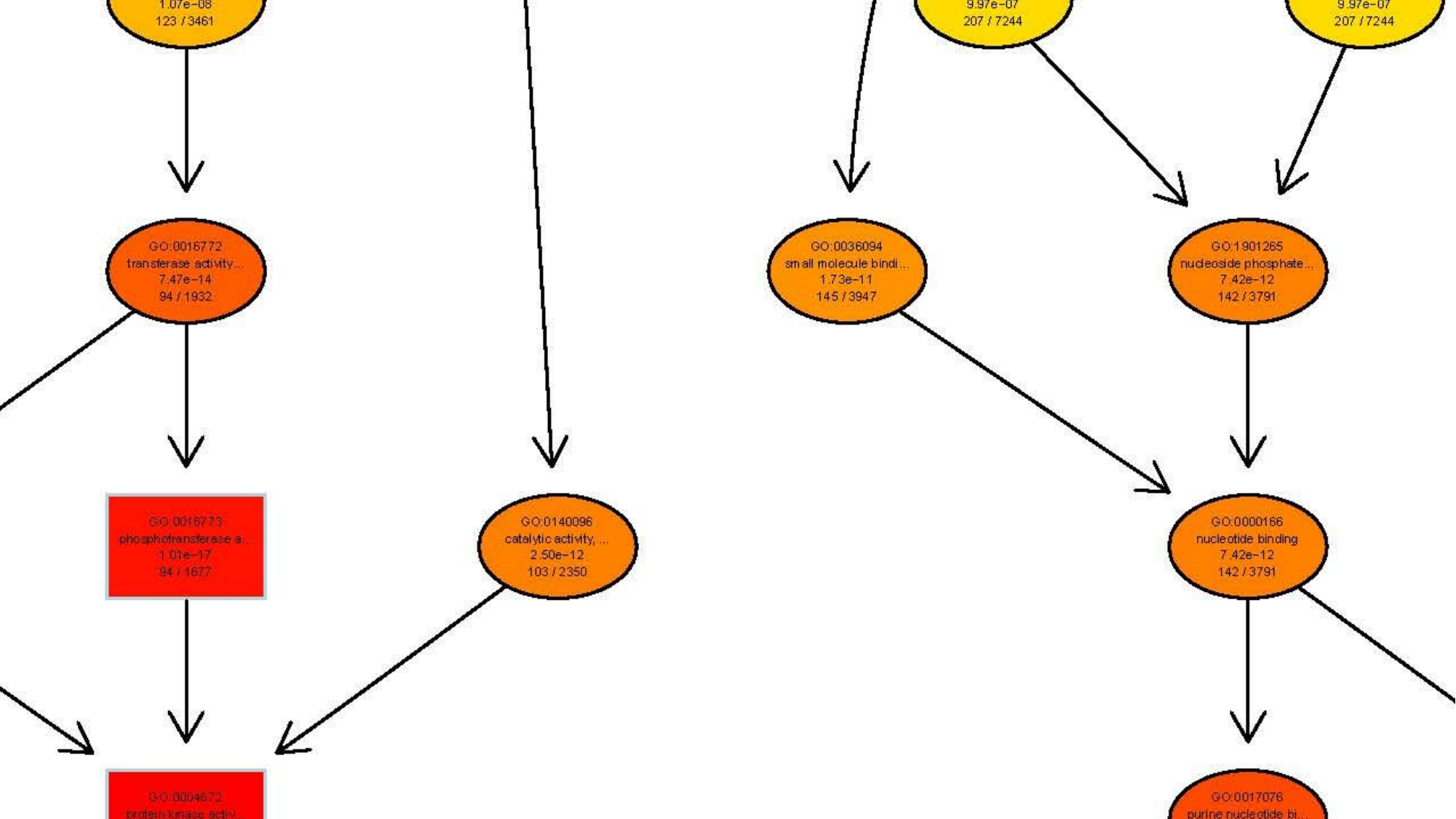

MF: Module 18
1
2
3
4
5
6
7
8
9
10
Modules : 38 white – 36 cyan – 26 turquoise – 22 Green – 18 black - 15 Yellow –
11
12
13
14
15
16
17
18
19
20
21
22
23
24
25
26
27
28
29
30
31
32
33
34
35
36
37
38

## Slide 5
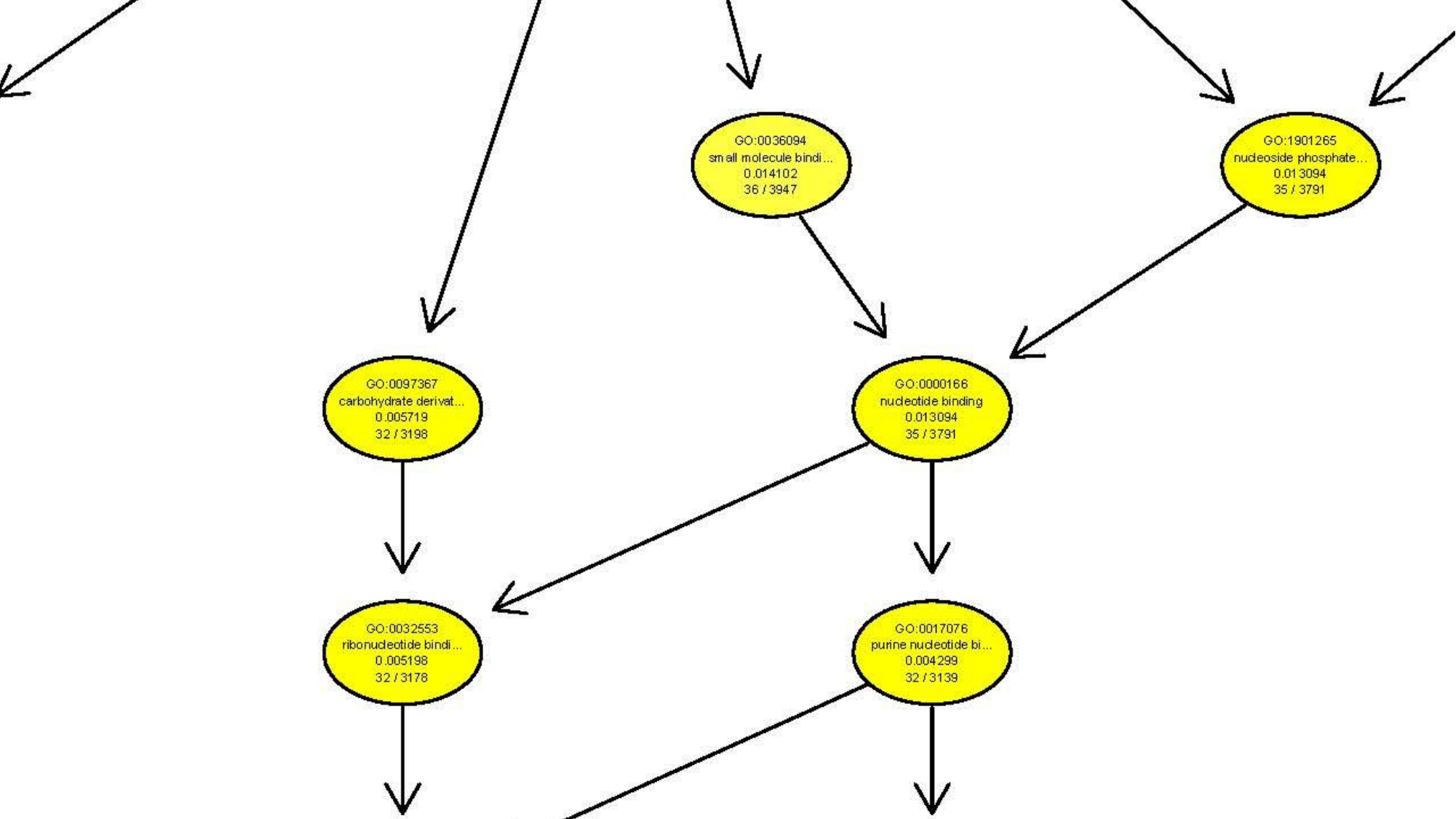

MF: Module 22

## Slide 6
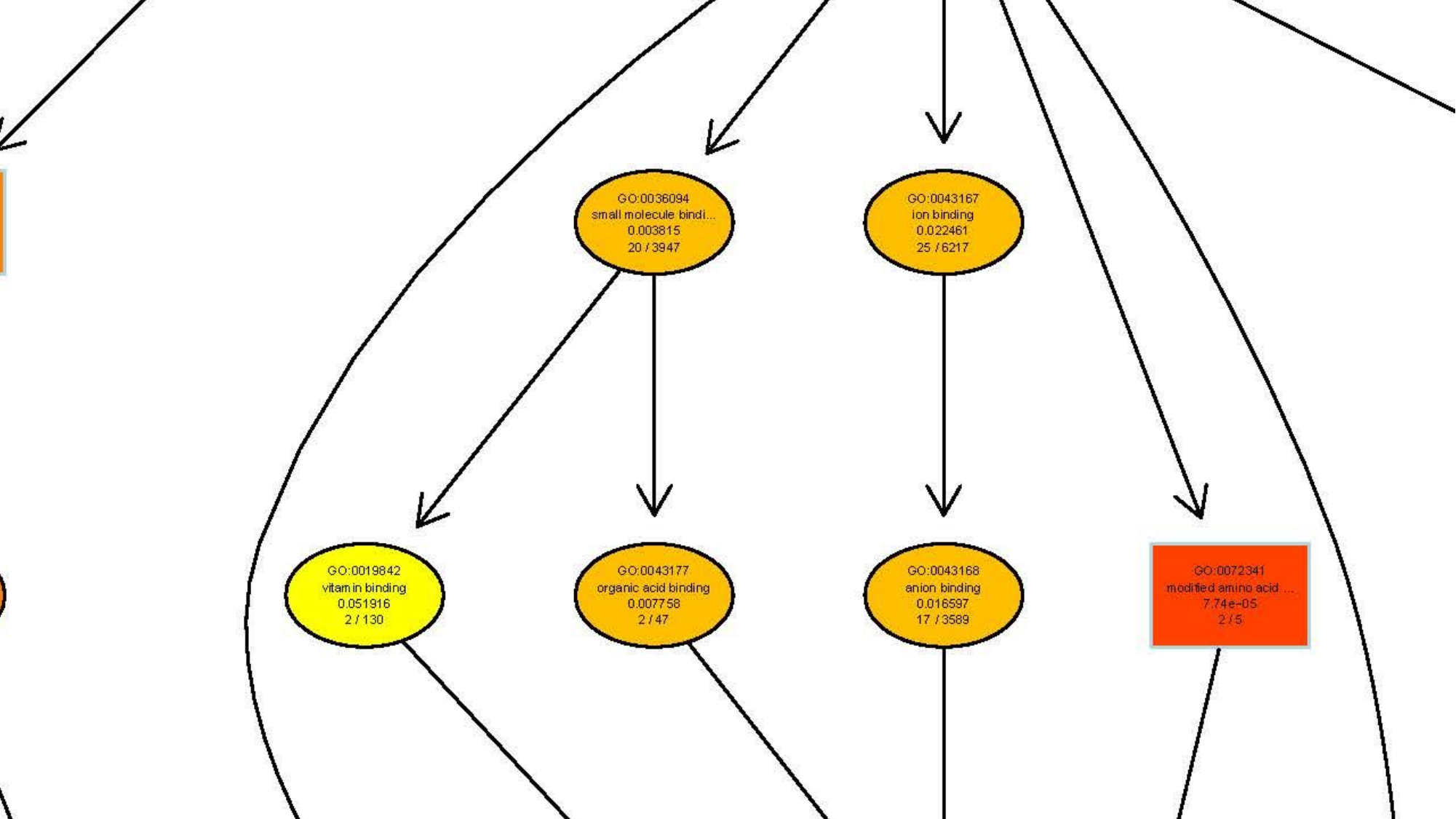

MF: Module 26

## Slide 7
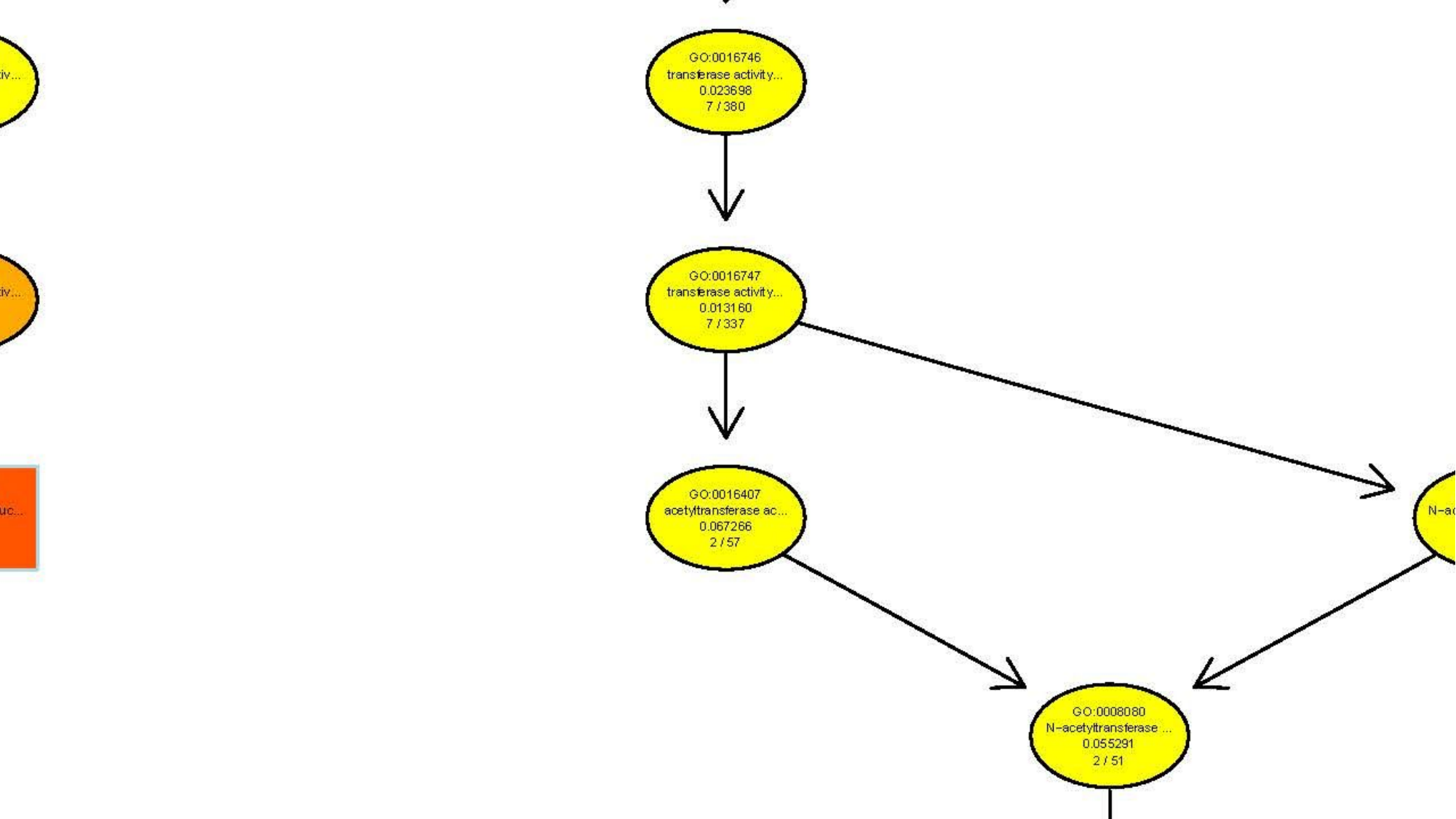

MF: Module 36

## Slide 8
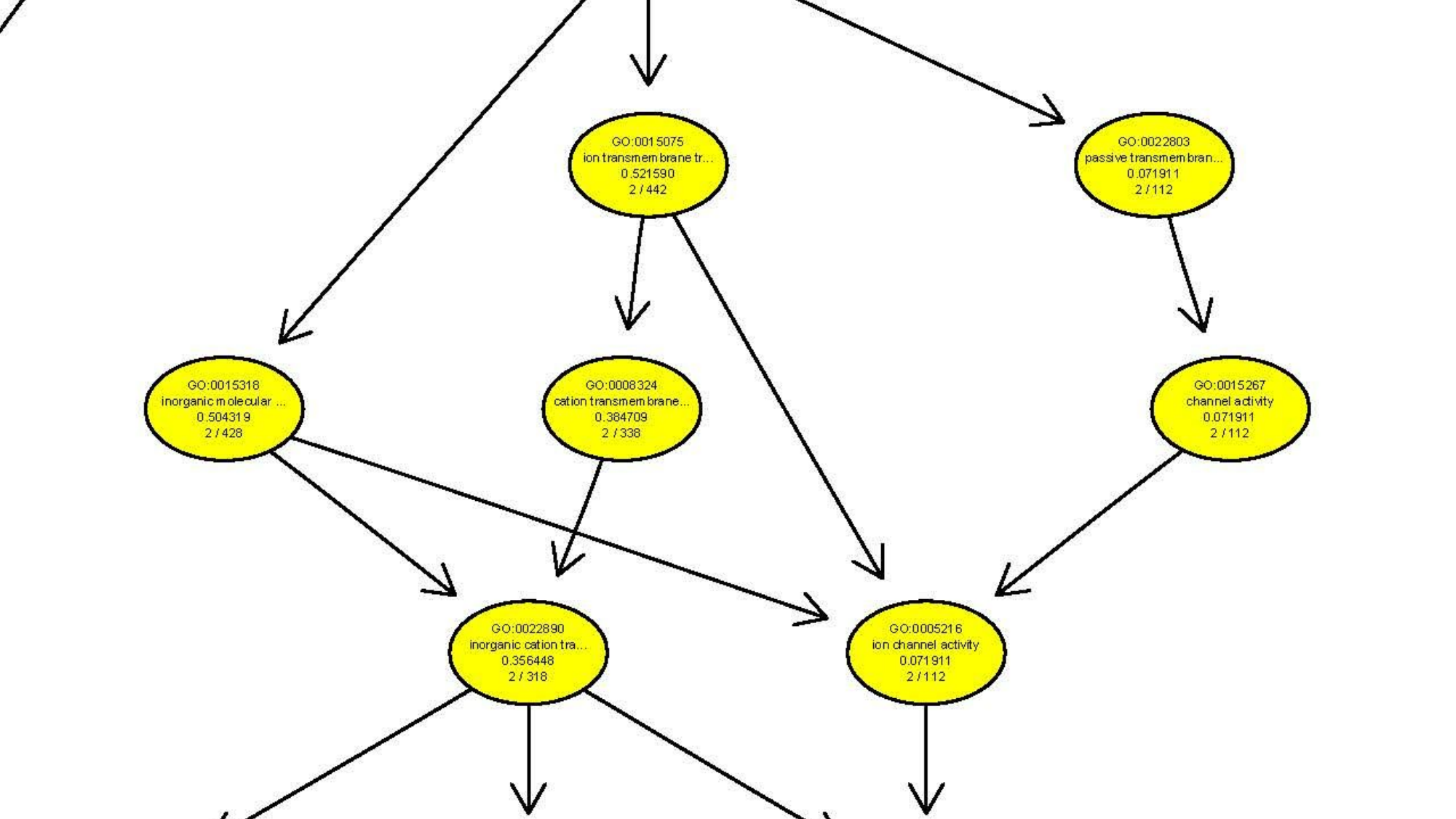

MF: Module 38

## Slide 9
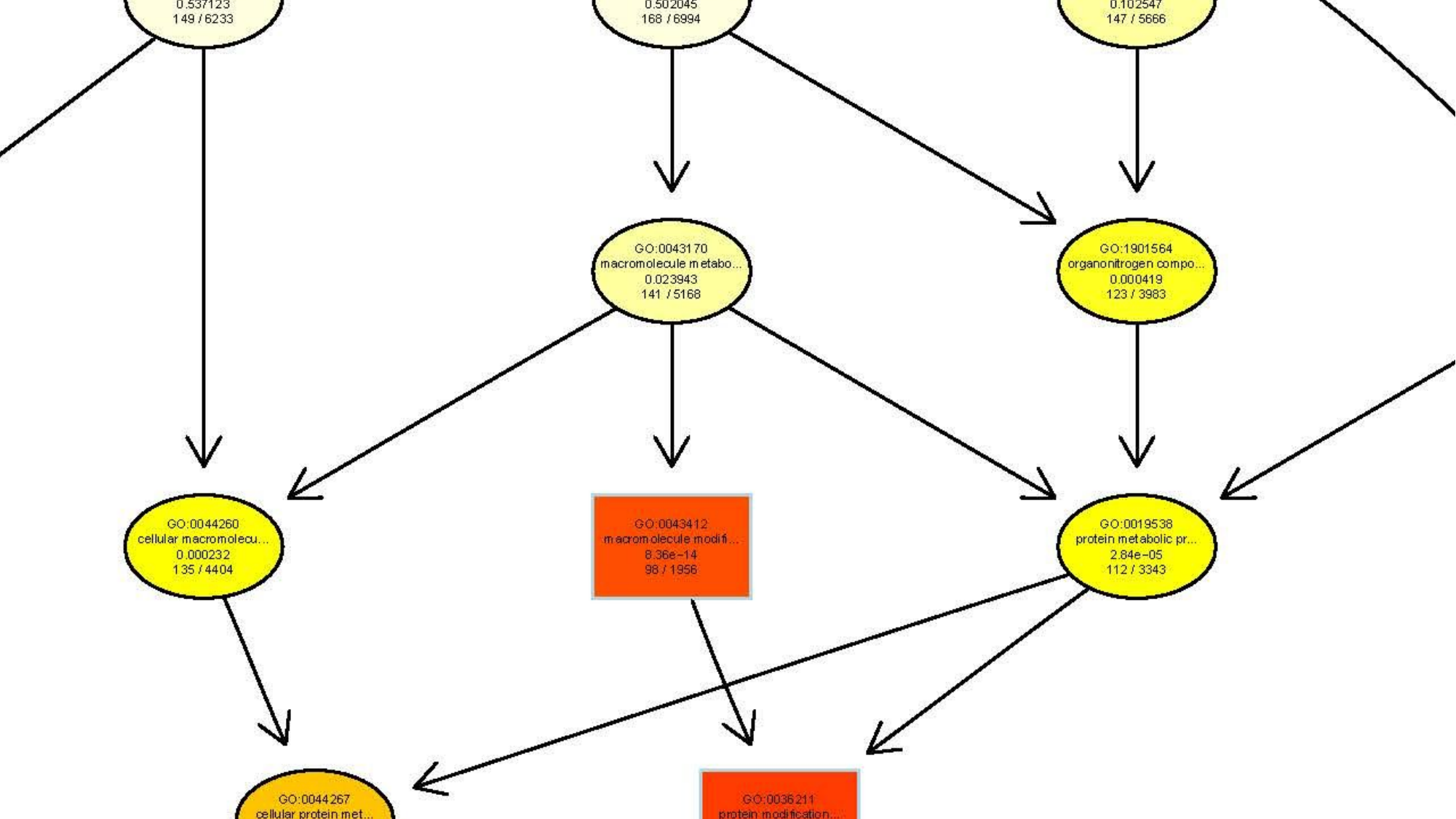

BP: Module 18

## Slide 10
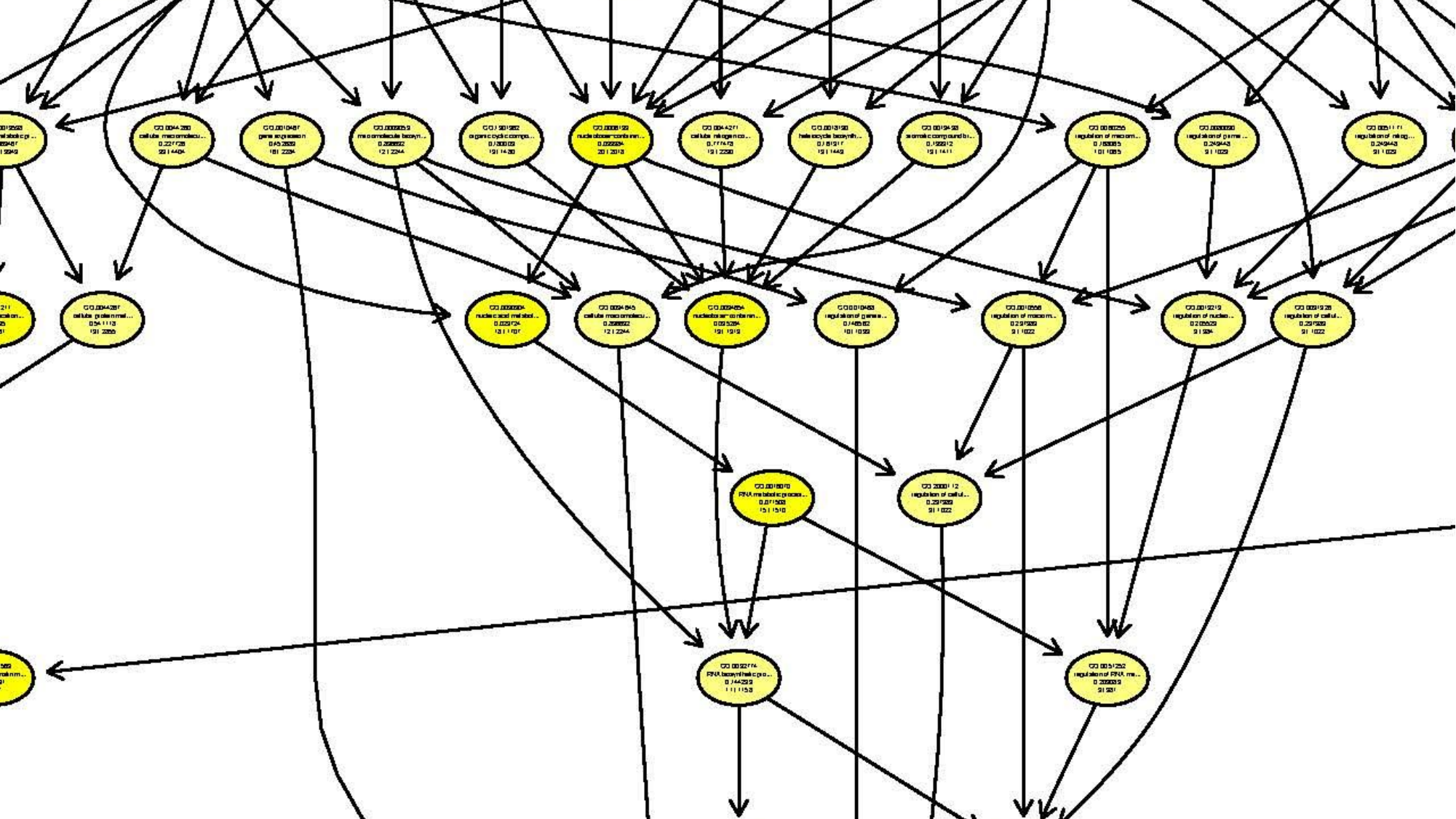

BP: Module 36

## Slide 11
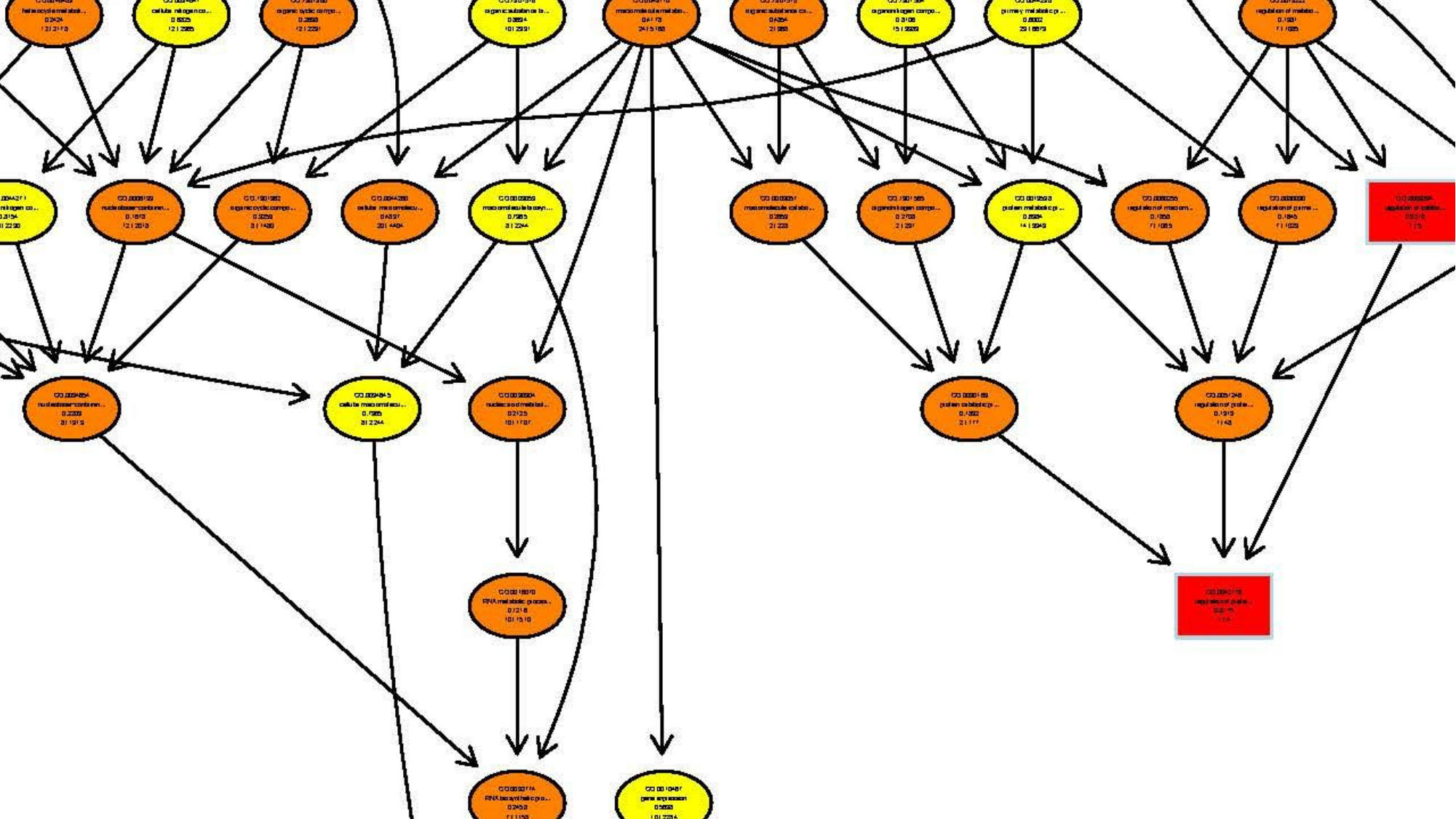

BP: Module 22

## Slide 12
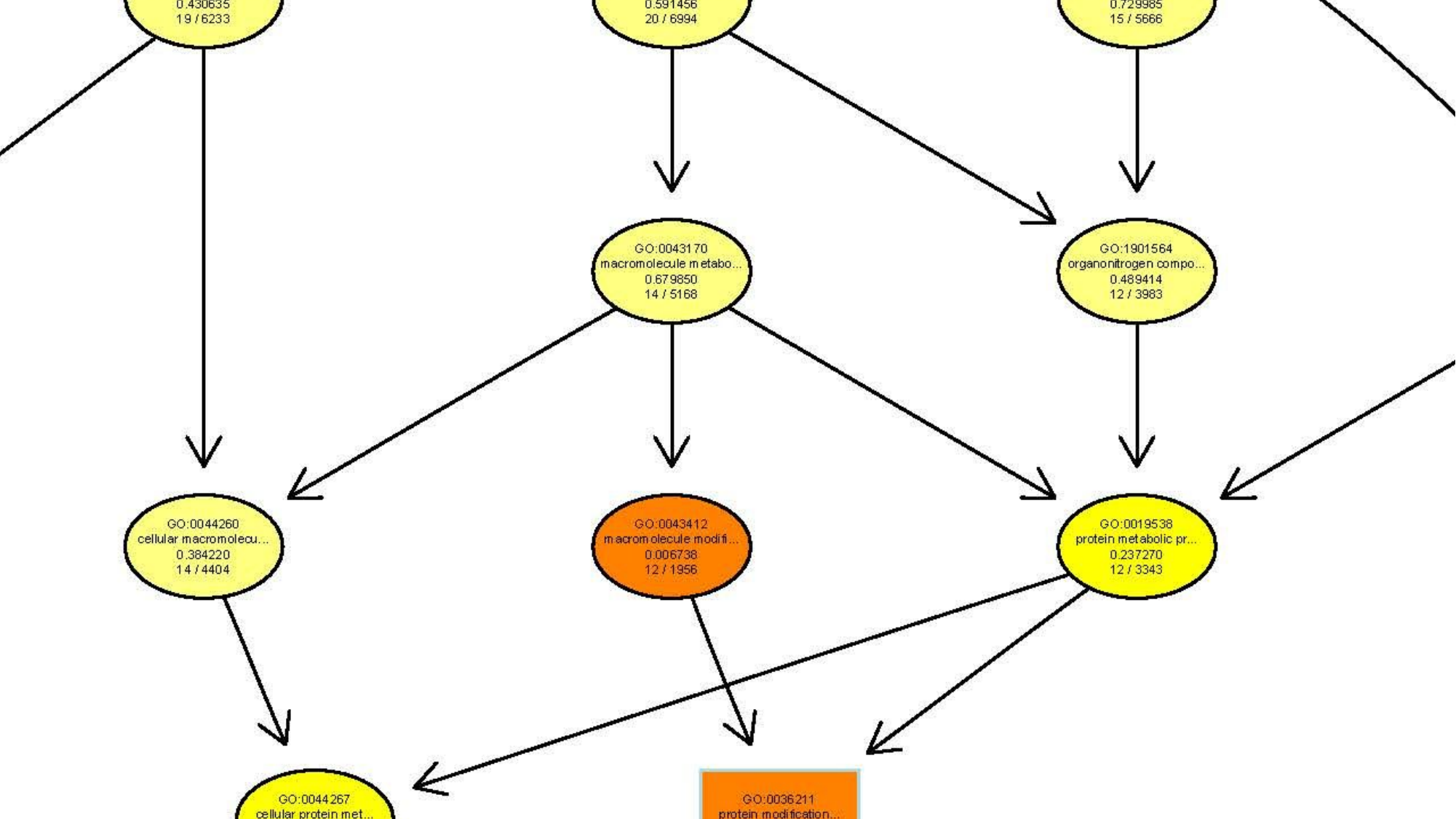

BP: Module 26

## Slide 13
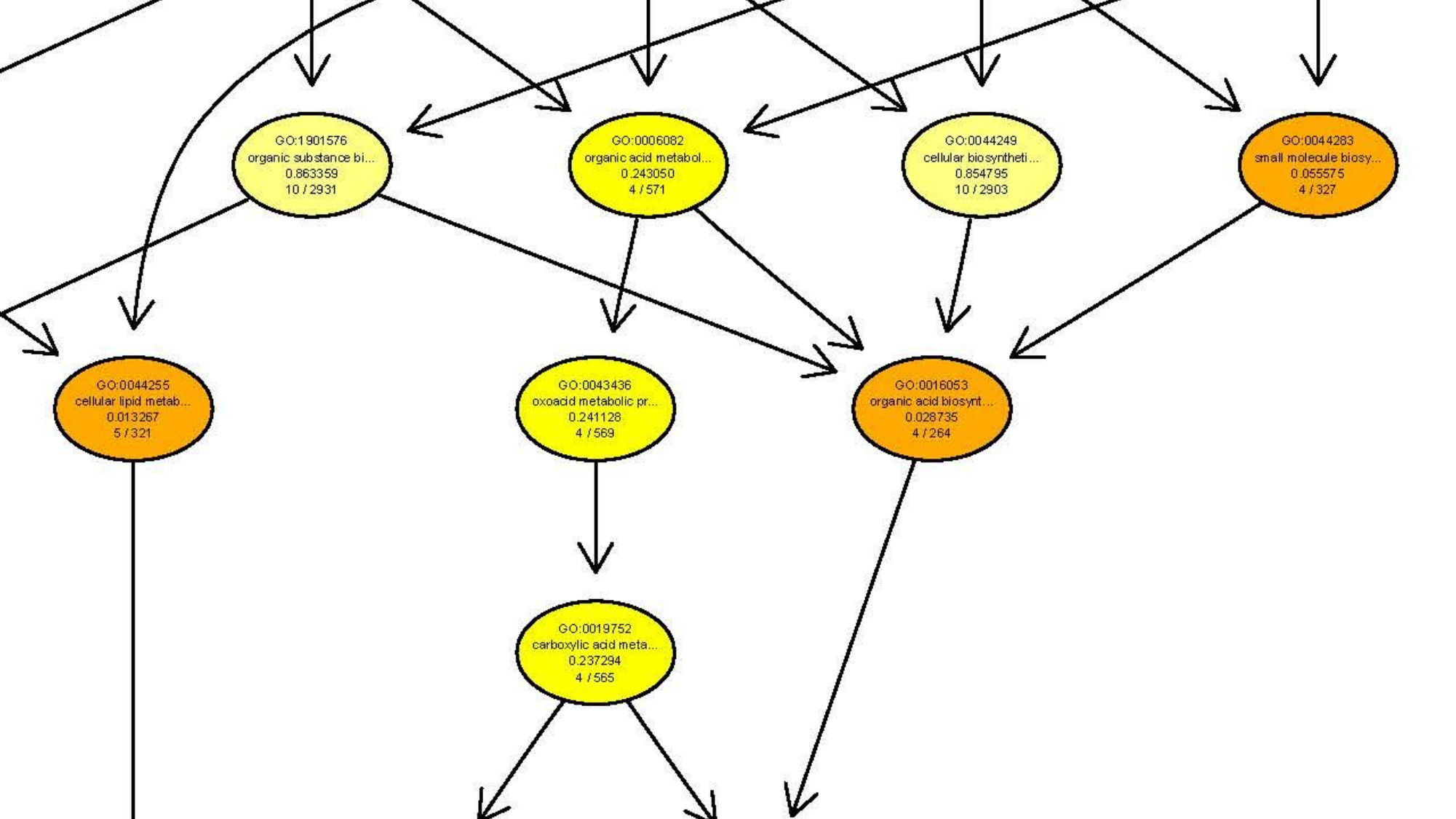

BP: Module 38

## Slide 14
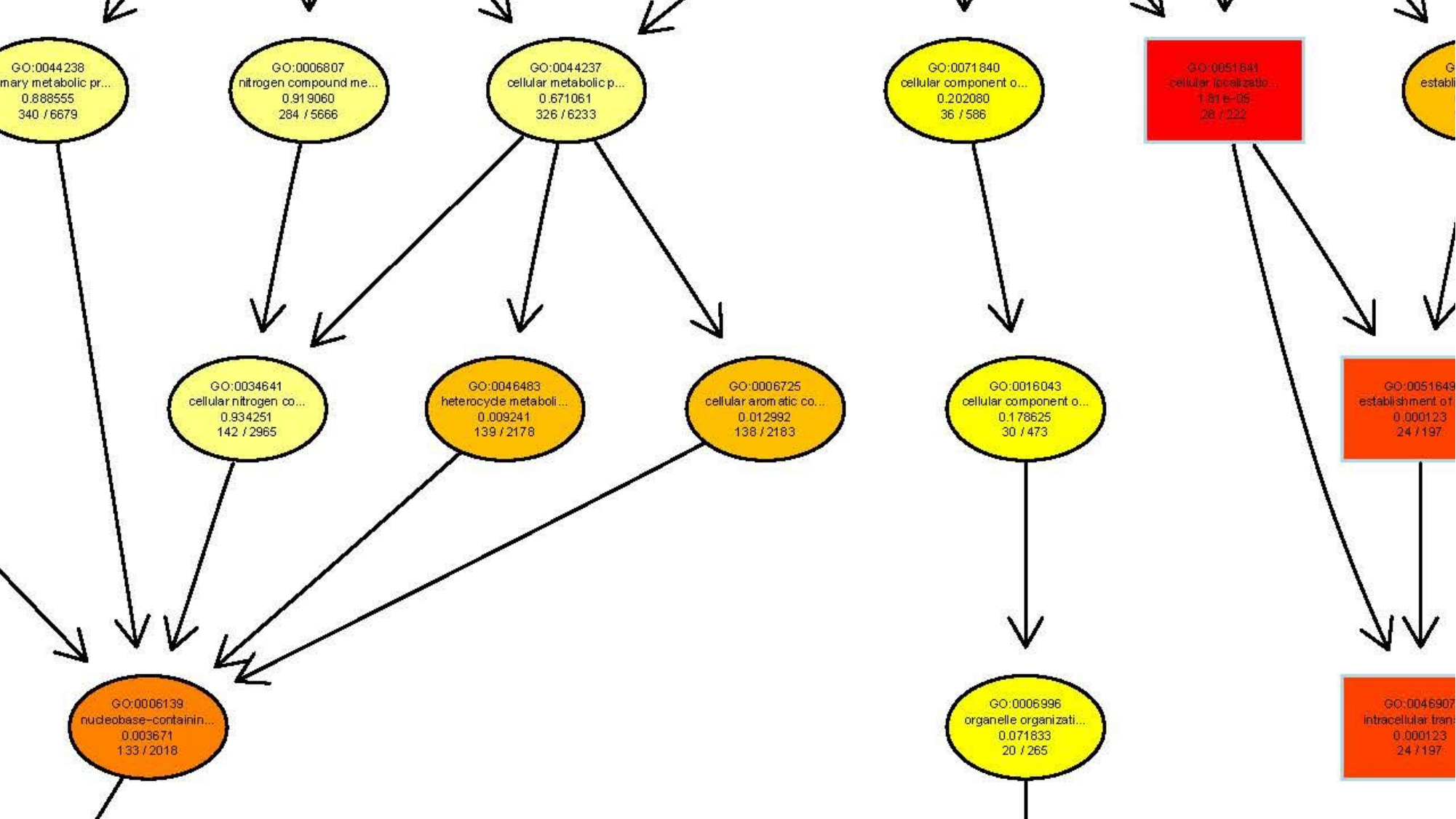

BP: Module 15

## Slide 15
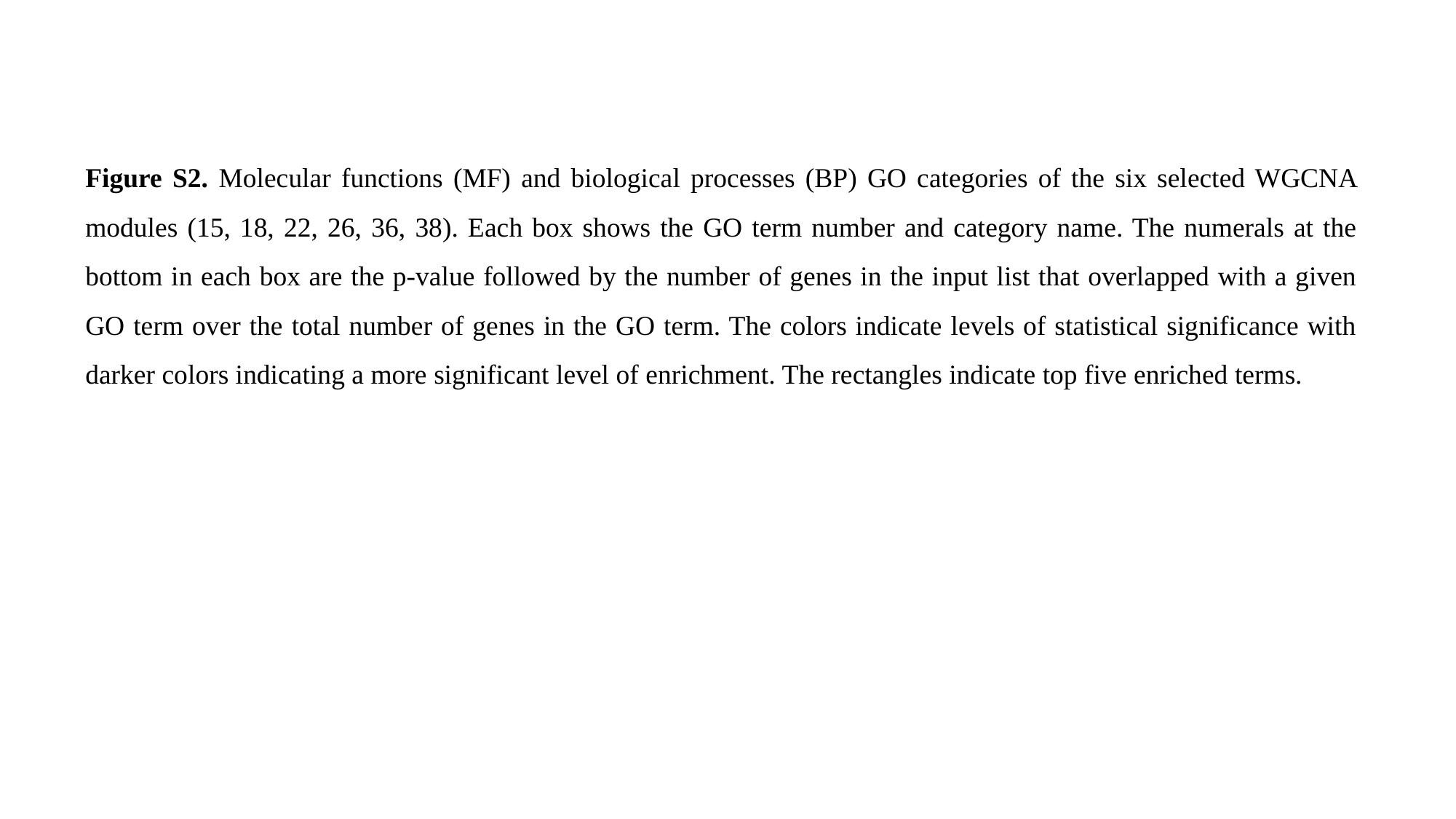

Figure S2. Molecular functions (MF) and biological processes (BP) GO categories of the six selected WGCNA modules (15, 18, 22, 26, 36, 38). Each box shows the GO term number and category name. The numerals at the bottom in each box are the p-value followed by the number of genes in the input list that overlapped with a given GO term over the total number of genes in the GO term. The colors indicate levels of statistical significance with darker colors indicating a more significant level of enrichment. The rectangles indicate top five enriched terms.
